# The Semmelweis Study: a longitudinal occupational cohort study within the framework of the Semmelweis Caring University Model Program for supporting healthy aging

**DOI:** 10.1007/s11357-023-01018-7

**Published:** 2023-12-07

**Authors:** Zoltan Ungvari, Adam G. Tabák, Roza Adany, György Purebl, Csilla Kaposvári, Vince Fazekas-Pongor, Tamás Csípő, Zsófia Szarvas, Krisztián Horváth, Peter Mukli, Piroska Balog, Robert Bodizs, Peter Ujma, Adrienne Stauder, Daniel W. Belsky, Illés Kovács, Andriy Yabluchanskiy, Andrea B. Maier, Mariann Moizs, Piroska Östlin, Yongjie Yon, Péter Varga, Zoltán Vokó, Magor Papp, István Takács, Barna Vásárhelyi, Péter Torzsa, Péter Ferdinandy, Anna Csiszar, Zoltán Benyó, Attila J. Szabó, Gabriella Dörnyei, Mika Kivimäki, Miklos Kellermayer, Bela Merkely

**Affiliations:** 1 International Training Program in Geroscience/Healthy Aging Program, Doctoral School of Basic and Translational Medicine/Department of Public Health, Semmelweis University, Budapest, Hungary; 2 Vascular Cognitive Impairment, Neurodegeneration and Healthy Brain Aging Program, Department of Neurosurgery, University of Oklahoma Health Sciences Center, Oklahoma City, OK USA; 3 Department of Health Promotion Sciences, The Hudson College of Public Health, University of Oklahoma Health Sciences Center, Oklahoma City, OK USA; 4 Department of Public Health, Faculty of Medicine, Semmelweis University, Budapest, Hungary; 5 UCL Brain Sciences, University College London, London, UK; 6 Department of Internal Medicine and Oncology, Semmelweis University, Faculty of Medicine, Budapest, Hungary; 7 HUN-REN-UD Public Health Research Group, Department of Public Health and Epidemiology, Faculty of Medicine, University of Debrecen, Debrecen, Hungary; 8 Institute of Behavioral Sciences, Faculty of Medicine, Semmelweis University, Budapest, Hungary; 9 Robert N. Butler Columbia Aging Center, Columbia University, New York, NY USA; 10 Department of Epidemiology, Columbia University Mailman School of Public Health, New York, NY USA; 11 Department of Ophthalmology, Faculty of Medicine, Semmelweis University, Budapest, Hungary; 12 Department of Ophthalmology, Weill Cornell Medical College, New York City, NY USA; 13 Department of Clinical Ophthalmology, Faculty of Health Sciences, Semmelweis University, Budapest, Hungary; 14 Healthy Longevity Translational Research Program, Yong Loo Lin School of Medicine, National University of Singapore, Singapore, Singapore; 15 Centre for Healthy Longevity, National University Health System, Singapore, Singapore; 16 Department of Human Movement Sciences, @AgeAmsterdam, Vrije Universiteit, Amsterdam Movement Sciences, Amsterdam, The Netherlands; 17 Ministry of Interior of Hungary, Budapest, Hungary; 18 WHO Regional Office for Europe, Copenhagen, Denmark; 19 Clinical Center, Semmelweis University, Budapest, Hungary; 20 Center for Health Technology Assessment, Semmelweis University, Budapest, Hungary; 21 Department of Laboratory Medicine, Faculty of Medicine, Semmelweis University, Budapest, Hungary; 22 Department of Family Medicine, Faculty of Medicine, Semmelweis University, Budapest, Hungary; 23 Department of Pharmacology and Pharmacotherapy, Faculty of Medicine, Semmelweis University, Budapest, Hungary; 24 Department of Translational Medicine, Faculty of Medicine, Semmelweis University, Budapest, Hungary; 25 HUN-REN-SU Cerebrovascular and Neurocognitive Diseases Research Group, Budapest, Hungary; 26 First Department of Pediatrics, Faculty of Medicine, Semmelweis University, Budapest, Hungary; 27 HUN-REN-SU Pediatrics and Nephrology Research Group, Semmelweis University, Budapest, Hungary; 28 Department of Morphology and Physiology, Faculty of Health Sciences, Semmelweis University, Budapest, Hungary; 29 Department of Biophysics and Radiation Biology, Faculty of Medicine, Semmelweis University, Budapest, Hungary; 30 Heart and Vascular Center, Semmelweis University, Budapest, Hungary

**Keywords:** Epidemiology, Healthy aging, Workplace cohort, Health Promoting University, Age-associated diseases, Central Europe, Biological age

## Abstract

The Semmelweis Study is a prospective occupational cohort study that seeks to enroll all employees of Semmelweis University (Budapest, Hungary) aged 25 years and older, with a population of 8866 people, 70.5% of whom are women. The study builds on the successful experiences of the Whitehall II study and aims to investigate the complex relationships between lifestyle, environmental, and occupational risk factors, and the development and progression of chronic age-associated diseases. An important goal of the Semmelweis Study is to identify groups of people who are aging unsuccessfully and therefore have an increased risk of developing age-associated diseases. To achieve this, the study takes a multidisciplinary approach, collecting economic, social, psychological, cognitive, health, and biological data. The Semmelweis Study comprises a baseline data collection with open healthcare data linkage, followed by repeated data collection waves every 5 years. Data are collected through computer-assisted self-completed questionnaires, followed by a physical health examination, physiological measurements, and the assessment of biomarkers. This article provides a comprehensive overview of the Semmelweis Study, including its origin, context, objectives, design, relevance, and expected contributions.

**Supplementary information:**

The online version contains supplementary material available at 10.1007/s11357-023-01018-7.

## Introduction

### Unsuccessful aging of the Hungarian population: a societal challenge

Population aging is a critical societal challenge in the European Union (EU) [1]. Currently, there are over 100 million inhabitants 65 years and older living in the EU and the UK. Their number is projected to increase to close to 150 million by 2050, which represents approximately 21.2% (2022) to 29.5% (2050) of the population of these countries [1, 2]. The achievement of sustainable development goals (SDGs), especially that of health-related SDGs strongly depends on the health status of aging populations, as the opportunities brought by increasing life expectancy can only manifest if the time lived with disabilities is compressed such that individuals go through a healthy (successful) aging process [3]. This brings new challenges for the health systems, including public health services, and society, as pursuing increased “healthspan” in addition to lifespan requires more emphasis on disease prevention and health promotion throughout life by involving all sectors of society and the people themselves.

Importantly, there are significant health differences between member states, which determine how successfully societies can adapt to these demographic changes. Hungary is a member of the EU and has a rapidly aging and declining population. In 2021, every fifth (20.3%) person was 65 or older [4]; that is projected to increase to 27.9% by 2050 [5]. The graying Hungarian society is facing complex demographic and public health challenges, which impact the health and social care systems, labor markets, public finances, and the pension system. From a public health perspective, it is essential to understand the drivers of functional disability attributable to age-related deterioration of health as well as the determinants of successful aging. Based on this information, governments can proactively implement public health policies and programs to enhance the well-being of their aging population and their participation in economic, social, cultural, and political activities.

Aging is the single most important risk factor for morbidity and mortality from a wide range of age-related, chronic non-communicable diseases [6]. It is increasingly recognized that the same fundamental biological mechanisms of aging are causally linked to the pathogenesis of diverse age-associated diseases, ranging from cardiovascular and neurodegenerative diseases to cancer. Rapid advances in the field of geroscience have identified evolutionarily conserved cellular and molecular processes (i.e., inflammatory mechanisms, endocrine regulation, nutrient sensing pathways, mitochondrial mechanisms, etc.) that underlie biological aging, regulate lifespan and contribute to the pathogenesis of virtually all age-associated diseases [7–9]. According to the concept originally proposed by Rowe and Kahn, “successful aging” (healthy aging) is characterized by high physical, psychological, and social functioning in old age and a lack of major age-associated diseases [10–12]. From a geroscience perspective, successful/healthy aging is defined by the optimization of biological aging processes throughout life and thereby minimizing the rate of age-related functional decline, which results in a delayed manifestation of age-associated diseases and maintaining longer the functional ability that enables well-being in older age (as the WHO defines “healthy aging” [13]). In contrast, unsuccessful/unhealthy aging is characterized by accelerated biological aging processes, resulting in a steeper trajectory of age-related functional decline and early onset of age-associated diseases. Individuals experiencing unhealthy aging are also more likely to have a lower socio-economic status, which has been associated with negative health outcomes, including premature manifestations of age-associated diseases [14]. This suggests that there is an equity aspect to healthy aging, where individuals from lower socio-economic backgrounds may face additional challenges in maintaining their health and well-being as they age.

The Hungarian population typically ages unsuccessfully as it is reflected by a range of epidemiological indicators. Although life expectancy increased since 1990, it was still at 75.7 years in 2020 (female 78.74, male 72.21 years), which was almost 5 years less than the EU average [15]. As a consequence of increased mortality during the COVID-19 pandemic the life expectancy decreased to 74.5 years in 2021 (female 77.5, male 70.7) [16]. Furthermore, the life expectancy of Hungarian people at the age of 65 is one of the lowest in Europe as well (women 18.6 years, men 14.8 years) [17]. Compounding the 2020 losses, life expectancy dropped significantly further throughout 2021, and by the end of 2021, the loss was 24.6 months in comparison with the 2019 figure [18]. According to OECD estimates, Hungary has the second worst age-standardized mortality due to preventable causes (243/100,000 population) based on data from 2019—almost twice the OECD average. The rate of treatable deaths (131/100,000 population) is the fourth worst among the OECD countries [15, 19, 20]. Regarding early mortality in 2019 premature (i.e., in the age group of 50–69 years), death rates caused by malignant diseases are the highest in Hungary for both sexes (males 741.13/100,000; females 432.86/100,000) among the EU countries [21].

Unsuccessful aging of the Hungarian population is also reflected by the low number of healthy life years, even compared to life expectancy. In 2020, a Hungarian man was expected to live healthy disease-free life until age 61.7, while a woman has 63.6 healthy life years [22]. Two-thirds of Hungarians who are at the age 65 years or older report having at least one chronic disease, which is 12 percentage points higher than the EU average [23]. Only less than 27% of older Hungarians consider themselves to be in good health, which is one of the lowest rates in the EU [1].

Age-associated chronic non-communicable diseases (including cardiovascular diseases, stroke, type 2 diabetes mellitus, malignancy, chronic respiratory diseases, low back pain, and mental health problems) account for over 85% of the disability-adjusted life years (DALYs) [24]in Hungary. Poor health is a major factor contributing to the fact that approximately 50% of older people (aged 75 years or more) have a low level of life satisfaction in Hungary, which rate is one of the highest among the member states of the EU [1].

Unhealthy aging is a major social and economic challenge for Hungary and one of the leading causes of impaired societal resilience (e.g., note the link between the socio-economic status and mortality observed in the second and third waves of the COVID-19 pandemic and the increased morbidity and mortality of the elderly) [25–28]. Due to their unhealthy aging, older citizens are at increased risk of psychological, social, economic, and biomedical problems related to old age, which is a serious obstacle to the creation of a safe society and the sustainability of economic growth. The economic inactivity of the unhealthily aging retirement-age population harms the national economy whereas successful aging can support economic development in many ways. Decreased dependency reduces the cost needed for social care of the elderly. Healthy, active, and productive elderly people, who continue paid or unpaid work beyond retirement age, make a positive social and economic contribution the society. Accumulation of asset wealth of successfully aging elderly and their private spending also benefit the economy [29–31]. Thus, investing resources and money in disease prevention of age-associated diseases and the promotion of healthy aging can yield substantial returns on investment, as demonstrated by the median return on investment and cost–benefit ratios reported in various studies [32]. This makes such investments highly attractive and advantageous for society, not only from a health perspective but also from an economic one.

The causes of the unhealthy aging of the Hungarian society are likely to be multifaceted and are not completely understood. Behavioral risk factors such as smoking, low physical activity, obesity, unhealthy diet, and hypertension account for half of all deaths in Hungary, well above the average of the European Union (39%) [15]. A quarter (26%) of Hungarian adults smoke regularly, and nearly one in three are obese—both above the EU averages [15]. Importantly, behavioral risk factors (such as smoking, low physical activity, unhealthy alcohol consumption, unhealthy diet) are often embedded in material, social, economic, environmental, and psychological conditions [33, 34]. For instance, smoking prevalence is often linked to material hardship, low social status, and other life stressors. Strong preclinical and clinical evidence support the concept that obesity as well as other lifestyle factors exacerbate cellular and molecular processes of aging, resulting in accelerated biological aging, which increases the risk for cancer, cardiovascular and metabolic diseases, and complex multimorbidity, as well as decreases in quality of life and accelerates cognitive decline [35–38]. It is expected that there are also a number of hidden or emerging risk factors that may also contribute to accelerated aging, promoting the manifestation and progression of age-associated diseases and resulting in high premature mortality of Hungarians.

### The Semmelweis Study: understanding determinants of healthy aging

Semmelweis University with over 12,000 students currently enrolled and located in Budapest, the capital of Hungary, is a highly regarded public research university renowned for its expertise in the fields of medicine and health sciences in Central Europe. The university’s mission is rooted in a dedication to innovative education, research, and healthcare services, with a particular emphasis on promoting excellence in cardiovascular medicine, diabetology, oncology, epidemiology, and public health across all levels of training, research, and practice. Semmelweis University is the largest provider of healthcare services in Hungary and is committed to providing national leadership in protecting and improving the health of its population.

To combat the adverse epidemiological situation in Hungary, the university is implementing a multidisciplinary Healthy Aging Program that spans preclinical, translational, clinical, and public health research with the goal of understanding determinants of unhealthy aging and developing pharmacological, nutraceutical, lifestyle, and other interventions to slow/optimize the aging process for the prevention of age-related diseases and ultimately extending healthy lifespan of the aging Hungarian population. As part of the epidemiological pillar of this research program, the Semmelweis University performed two population-based surveys of cardiovascular risk factors, the Budakalász Health Examination Survey [39, 40] and the H-UNCOVER survey [41]. The first aimed to enroll the adult population (> 20 years, ~ 8000 inhabitants) of a Central-Hungarian town, Budakalász. The H-UNCOVER study was a cross-sectional survey in a representative sample (*n* = 17,787) for the adult Hungarian population to estimate the rate of active infection and the prevalence of prior SARS-CoV-2 exposure during the first wave of the COVID-19 pandemic. A secondary goal was to define the prevalence of important risk factors for cardiometabolic diseases, and that of chronic non-communicable diseases (NCDs) [41]. While these population-based surveys are able to estimate the prevalence of NCDs and their risk factors in Hungary, they were not designed to examine the temporal relationship between risk factors (including novel biomarkers) and disease outcomes as well as determinants of healthy aging [42].

Inspired by the success of the milestone workplace/occupational cohort studies of the twentieth century, including the Whitehall II study, the British Doctors’ Study [43, 44], the Nurses’ Health Study [45], and others, we have conceived the Semmelweis Study as a prospective workplace cohort study of all employees of the Semmelweis University. Occupational and workplace-based cohorts, comprising employees from various facilities within a given industry (e.g., members of a professional association) or individual facilities and organizations have made significant contributions to our understanding of occupation-related exposures [46] and the relationships between employment (e.g., shift work [47]) and health [46]. In the past decades, occupational and workplace-based cohorts have been designed to investigate an increasingly broadened spectrum of health outcomes [48], which resulted in the identification of a wide range of social, lifestyle, environmental, and biological risk factors that promote the development and progression of various chronic diseases.

The Semmelweis Study takes inspiration in particular from the Whitehall studies. The original Whitehall study was designed as a longitudinal workplace cohort study investigating cardiorespiratory disease and diabetes mellitus, looking at individual risk factors for disease in a population of middle-aged men employed by the British Civil Service. It soon became evident that socioeconomic differences in the civil service are strong predictors of both physical and mental illnesses and mortality [49]. Based on these findings, the Whitehall II study was then set up to examine the contributions of the social gradient to health and disease, extending the investigation to include women. In Whitehall II, men and women have been followed-up for over 35 years. with repeated clinical examinations and linked electronic health records, and with cohort maturation, it has become an important study of aging (dementia, frailty, disability, etc.). Regarding job characteristics, major coronary heart disease incidence was highest in those who reported high job strain [50], a finding subsequently confirmed in large-scale individual-participant meta-analyses [51].

Whitehall II was one of the first studies to describe biomarker trajectories leading to diabetes diagnosis, including the changes in glucose concentrations, insulin sensitivity, and insulin secretion [52]. It has also shown that “metabolically healthy obesity” (i.e., obesity without metabolic risk factor clustering) is a transitory state progressing toward glucometabolic abnormalities (rather than a stable phenotype [53]) and that alcohol consumption, even at moderate levels, is associated with adverse brain outcomes including hippocampal atrophy [54]. Accordingly, findings from the Whitehall II study have been featured in several clinical guidelines and policy documents [55–57].

To move this field of research forward, the overall goal of the Semmelweis Study is to identify novel etiological mechanisms and risk factors for cardiometabolic, mental, cognitive, and malignant diseases as well as all-cause and cause-specific mortality, successful aging, and frailty with a special interest in socio-economic, psychological, lifestyle, environmental determinants, and biological markers. The results of the cohort study could contribute to the foundation of general and personalized interventions supporting the successful aging of working populations. The effect of interventions addressing these factors could be monitored over time using epidemiological methods and tested in randomized controlled trials within the caring university framework. In the following section, we describe the aims, objectives, design, data collection, analysis, and operational strategies of this novel workplace cohort study.

## Aims and objectives of the Semmelweis Study

The Semmelweis Study has been conceived to investigate the mechanistic links between lifestyle, environmental and occupational risk factors, and development and progression of chronic age-associated diseases. The study aims to identify groups of people who are aging unsuccessfully and therefore have an increased risk for such diseases [58]. We envision that the results of this cohort study targeting university employees may assist universities in supporting their employees to live the longest and healthiest life possible by identifying actions to promote health and well-being for all.

The Semmelweis Study consists of a baseline examination and 5-year follow-up examinations with assessments similar to the baseline examination. In addition, using data linkage to administrative databases, we plan to collect follow-up data regularly (every 2 years) on medication use, hospitalizations, sick leave, and social benefits. The following long-term objectives are defined:

(I) To characterize the health status and health determinants of Semmelweis University staff by defining the prevalence of cardiometabolic, neuropsychological mental/behavioral disorders, musculoskeletal diseases, neoplastic diseases, and risk conditions
(II) To describe causal and non-causal risk factors and health determinants of incident chronic diseases and mortality, such as health behaviors including diet, physical activity (especially during leisure time) and lifestyle factors, sleep hygiene and sleep–wake cycle, psychological well-being, determinants and competencies of resilience and self-efficacy, stress-management skills, and protective determinants which can be developed or strengthened (e.g., mental flexibility, resilience, self-motivation)
  - This includes an assessment of the gap between the chronological and biological age to identify the biological, physical, and psychological determinants of healthy aging and late-life flourishing.
  - The complex relationships among psychosocial, behavioral, and environmental risk factors, biological age, and the incidence of age-associated diseases, such as cardiovascular disease (CVD), cancer, metabolic diseases, depression, and other outcomes will be analyzed.
  - Molecular biology and laboratory analyses of biospecimens will be performed to elucidate mechanisms contributing to accelerated aging phenotypes.
  - Additional nested case–control and case-cohort studies using stored biospecimen to determine novel promising biomarkers with the optimal use of available resources will be performed.
(III) Semmelweis Study using repeat clinical examinations and sample collections will assess changes in health status and health determinants to facilitate understanding the natural history of diseases and aging through the investigation of trajectories of these markers.
(IV) The use of standardized data collection allows comparisons with general population studies if the same indicators are available from census and/or health examinations or interview surveys. It also facilitates international comparisons with similar occupational cohorts such as the Whitehall II study.
(V) Through data sharing agreements, results of Semmelweis Study participants can be included in international consortia that aim to investigate rare outcomes (where our and other cohorts by themselves have insufficient power) or more frequent outcomes but with much more precision.
(VI) The study could assist in identifying at-risk populations that could benefit from targeted health promotion and prevention interventions, including those based in the workplace. By identifying specific groups who may be at higher risk for certain health conditions, interventions can be tailored to address their unique needs and circumstances, potentially leading to more effective and efficient prevention efforts. Furthermore, workplace-based interventions have the potential to reach a large number of individuals and may have a significant impact on improving health outcomes for employees. Collecting data before and after the implementation of interventions can help to investigate the long-term effectiveness of these measures, which can complement the short-term quality control studies.

The specific objectives for the first 5 years of the study (the part that has funding secured) are to quantify the effect of socio-economic, psychological, lifestyle, and environmental determinants and biological markers on metabolic, cardiovascular, psychological, and psychiatric diseases, as well as mortality (Table 1).

**Table 1:** Specific endpoints of the study

Primary endpoints:	Obesity, metabolic syndrome, T2DM, hypertension, hyperlipidemia, arteriosclerosis
Secondary endpoints:	Other cardiovascular diseases
Tertiary endpoints:	Cognitive impairment, dementia
Further endpoints:	Burn-out, musculoskeletal problems, depression

An extremely important result of the Semmelweis Study is that by providing weights for different risk factors of chronic diseases, it will enable recalibration of generally used risk calculators tailored to the Hungarian population. These calculators will help clinicians estimate risks for individual patients and could also be used to determine target populations and priorities for health promotion programs. The results could be also used to model the outcomes of different intervention programs.

A deeper understanding of the aging process at genetic, molecular, organ, and system levels, and identifying the biological, physical, and psychosocial factors of successful aging will contribute to the development of national programs for healthy aging. A potential unique outcome of the study is the input for the development of a comprehensive proposal on how employees can optimize their physical and cognitive aging, thereby improving wellbeing and quality of life and increasing their healthy life expectancy. The Whitehall study demonstrated that the social gradient within the civil service is associated with inequalities in health and the incidence of chronic diseases [59–67]. Given the differences between the social structure of the UK and Hungary and the potentially larger variability in wealth and social status within Hungary, we expect that the Semmelweis Study will reveal previously unknown factors determining the social gradient in morbidity and mortality, which are specific to Hungary and Central Europe. A long-term aim of the Semmelweis Study is to identify the mechanisms that contribute to inequalities in biological aging, cardiovascular and cerebrovascular diseases, and diabetes mellitus in the investigated population.

## The Semmelweis Study within the framework of the “Semmelweis Caring University” model program of Semmelweis University

The leadership of the Semmelweis University has realized the very complex public health challenges of Hungary and the lack of a comprehensive national program to address the unhealthy aging of its population. Guided by the Okanagan Charter [68], it decided to develop a new model program based on the “Health Promoting University” initiative, adapted to a health sciences university environment. The Semmelweis “Caring University” model program is designed to improve the health and promote the well-being of all the university’s employees using cutting-edge approaches to public health and preventive medicine [42]. The “Caring University” model program also serves as a pilot project for national health promotion/disease prevention programs implemented in occupational settings.

The “Caring University” model program provides a university-wide context to the Semmelweis Study. Semmelweis University adopted a conceptually new approach and created a multifunctional and multi-level service center (Center of Preventive Services). The goal of the Center of Preventive Services is to provide integrated preventive services, including health promotion programs and lifestyle counselling based on health status and medical risk assessment. The Semmelweis Study was conceived to be an integral part of the “Caring University” model program from its start. First, the description of the health status of the employees provides data for the planning of the size of preventive services. Furthermore, with the identification of the risk factors with the most population-attributable risk, it helps the planning of the actual services to reach the most health gain for the employee population. Second, as the study aims to explore social determinants of health, it could provide important input to promote the health equality of university workers. Third, given the planned repeat 5-year assessments of the Semmelweis cohort, it could provide long-term efficiency data for the Center of Preventive Services.

The Semmelweis Study was initiated as a collaborative effort of the Department of Public Health, the Institute of Behavioral Sciences, the Center for Health Technology Assessment, the Faculty of Health Sciences, and the Heart and Vascular Centre of the University with additional support from experts at the Department of Epidemiology and Public Health at University College London, the Department of Epidemiology at Columbia University Mailman School of Public Health and the Department of Health Promotion Sciences at the Hudson College of Public Health, University of Oklahoma Health Sciences Center on specific aspects such as health inequality and assessment of biological age and cognitive function in 2021. The Semmelweis Study also benefits from input from the World Health Organization (WHO), which is leading the implementation of the United Nations Decade of Healthy Ageing (2021–2030) in collaboration with other UN organizations and serves as the Decade Secretariat [69]. This global collaboration aims to improve the lives of older people, their families, and the communities in which they live, and the Semmelweis Study aligns with this mission by investigating the factors that contribute to healthy aging and identifying interventions that can improve the health and well-being of older adults.

## Methods

### Design and sample

The Semmelweis Study is planned to be a prospective occupational cohort study. It intends to build on the experiences of the British Whitehall II study conducted by University College London [49]. Semmelweis Study will comprise a baseline data collection with an open healthcare data linkage followed by repeated data collection waves every 5 years.

**Fig. 1:**
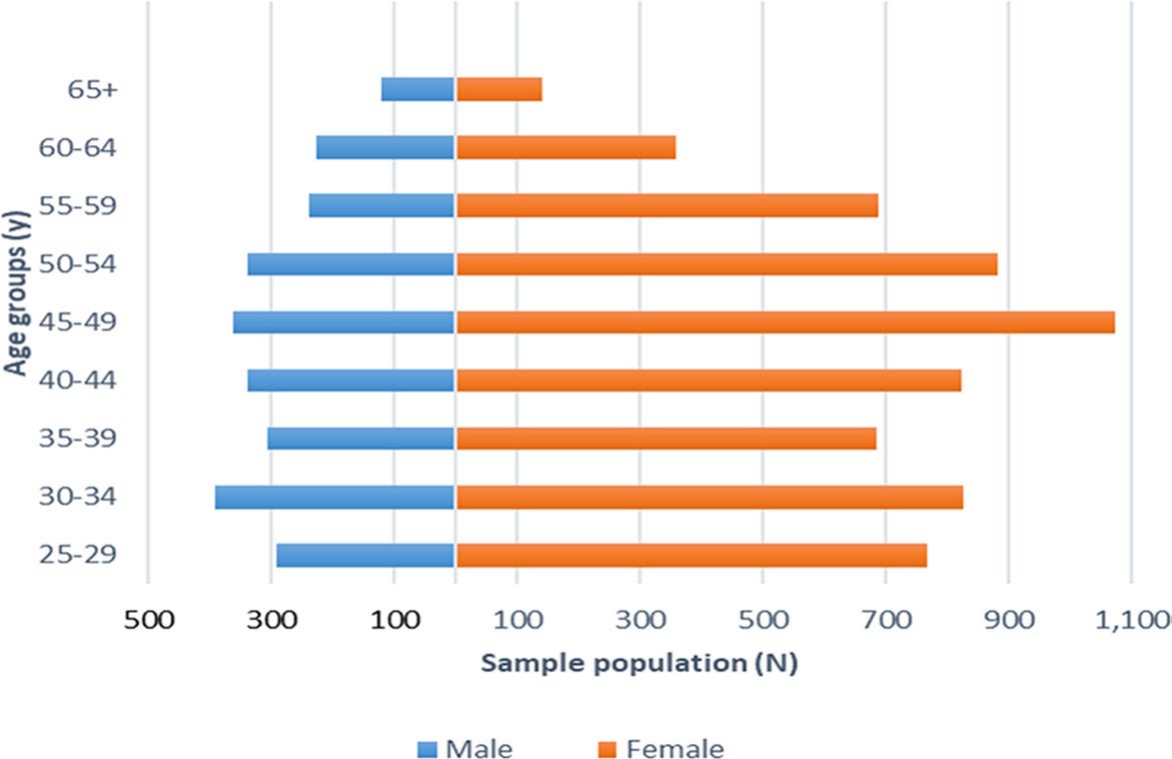
Age distribution of the target sample, the employees of Semmelweis University. Aggregated data were provided by the Semmelweis University HR Department

The study aims to enroll all employees of Semmelweis University aged 25 years and older, a population of 8866 people (at the time of the writing of this summary), 70.5% of whom are women. There are no exclusion criteria for the questionnaire; however, some of the laboratory or other physiological examinations have specific exclusion criteria. For example, no oral glucose tolerance test is performed in persons with known diabetes or a fasting glucose > 7 mmol/l (based on point of care glucometer). Furthermore, we exclude all known pregnant women from the physiological and laboratory examinations. The participation rate in the baseline data collection is expected to be around 70%, taking into account the baseline participation rates of similar occupational cohorts conducted in Europe (e.g., GAZEL [70], the Helsinki Health Study [71], the Whitehall II study [49], the Finnish Public Sector study) [72], particularly in healthcare settings (e.g., WHALE study) [73] while also considering the response rates in previous population health surveys in Hungary [74, 75]. The most numerous age groups in the target sample are women 45–49 and men 30–34 years of age (Fig. 1).

Given that there are over 100 occupational positions listed for the employees of Semmelweis University, it is expected that several aspects of work characteristics (including socioeconomic status) can be investigated (Table 2). To facilitate international comparisons, occupational data will be translated into the widely used International Standard Classification of Occupations (ISCO) developed by the International Labour Organization (ILO).

**Table 2:** Occupational characteristics of Semmelweis University employees in the target sample (aged ≥ 25). Aggregated data were provided by the Department of Human Resources of Semmelweis University

	Female (%)	Male (%)	Total (%)
	*n* = 6247 (70.5%)	*n* = 2619 (29.5%)	*n* = 8866 (100%)
Age (SD)	44.0 (11.0)	44.5 (11.9)	44.1 (11.3)
Occupation			
Healthcare	3462 (55%)	915 (35%)	4377 (49%)
Academic	843 (13%)	813 (31%)	1656 (19%)
Administrative	1357 (22%)	318 (12%)	1675(19%)
Manual	344 (6%)	453 (17%)	797 (9%)
Other	241 (4%)	120 (5%)	361 (4%)

While employees will be contacted through the use of university-provided e-mail addresses and mobile phones for the first wave of data collection, we will collect personal e-mails, addresses, and phone numbers as contact information for later waves. This will allow tracking participants retiring or moving to other employers. Awareness will be also enhanced by university-wide information campaigns, including newsletters and targeted departmental meetings. After the goal and structure of the study are presented, employees will be invited to voluntarily participate in the study. Forms of informed consents for data collection and passive data linkage with healthcare data from the National Health Insurance Fund will be collected at the baseline assessment. The baseline data collection is expected to start in the second quarter of 2023.

### Baseline data collection and derived variables

#### Questionnaire-based data collection

At baseline, a computer-assisted personal interview is completed including a self-completed questionnaire on the most important socio-economic and health-related factors (i.e., lifestyle factors, mental health status). The survey has been compiled specifically for the study comprising of questionnaires that are well-accepted and validated internationally and also in Hungary (Table 3; Supplement 1).

**Table 3:** Questionnaire domains at baseline assessment

Factors	Source of questionnaires/items
Sociodemographic factors	EHIS 2019 Hungarian version [76]
Job security	Copenhagen City Heart Study [202]
Family history	Whitehall II [203]
Medications	EHIS 2019 Hungarian version [76, 204]
Health status/diseases	EHIS 2019 Hungarian version [76, 204] VAS of EurQoL [205]
Quality of health	SF-36 [206]
Smoking, e-cigarettes	Heaviness of Smoking Index [207]
Alcohol consumption	AUDIT-10 [208]
Physical activity	IPAQ Short Form [209]
Sleeping quality	Pittsburg Sleep Quality Index [102]
Oral health	WHO Oral Health Questionnaire for Adults [210]
Nutrition	EHIS 2019 (Hungarian version)
Cognitive function	ICAR-5 [105, 107]
Stress	Perceived Stress Scale [88, 89]
	Effort-reward imbalance [90, 91]
	Social support [99, 100]
	Maslach burnout inventory [211]
Quality of relationships	Relationship structure [97, 98]
	Marital stress [93, 94]

Abbreviations: *AUDIT-10*, 10-item Alcohol Use Disorders Identification Test; *EHIS*, European Health Interview Survey; *EurQoL*, European Quality of Life Scale; *ICAR-5*, 5-item International Cognitive Ability Resource test; *IPAQ Short Form*, Short form International Physical Activity Questionnaire; *SF-36*, 36-Item Short Form Survey; *VAS*, visual analog scale

A main component of the questionnaire comes from the European Health Interview Survey (EHIS) wave 2 and consists of four modules: (a) health status, (b) healthcare use, (c) health determinants, and (d) socio-economic variables. The health status module covers self-perceived health, chronic diseases known by the respondents, limitations in activities, and mental health. The healthcare use module covers the use of different types of healthcare services, including hospitalizations, consultations, preventive (among them screening) services, and medications, and unmet needs for healthcare. The health determinants module collects data on smoking and alcohol consumption, physical activity, and dietary habits. Background variables on demographics and socio-economic status will be collected, including age, sex, education, living conditions, education, employment, occupation, and income [76].

Psychological factors are important correlates of health and productivity and poor psychological health is associated with several physical health conditions [72, 77–81]. The Job-Demand-Control-Support model by Karasek and Theorell is a useful tool for examining the impact of job characteristics on employees’ psychological well-being and physical health [82]. This model highlights how job demands, such as heavy workload and role ambiguity, can lead to stress for employees. The model suggests that individuals can mitigate these stressors by utilizing job skills that promote autonomy and control. Studies have shown that high demand and low control in the workplace are associated with various health issues, including cardiovascular and mental disorders [82–87]. However, social support at work can alleviate the negative effects of these working conditions. Therefore, a comprehensive set of inventories and questionnaires will be used in the study. Overall psychological well-being will be assessed by SF-36 and EuroQoL. Daily stress will be assessed with the Perceived Stress Inventory. The Hungarian version of the 10-item Perceived Stress Scale (PSS10) will be used to measure the participants’ perception of stress [88]. The PSS10 was designed to measure “the degree to which individuals appraise situations in their lives as stressful” [89]. The items evaluate the degree to which people find that life is unpredictable, uncontrollable, or overloaded. As the questions are quite general in nature, they are relatively free of content specificity to any population subgroup. As the stress/coping balance is a better predictor of psychological status than stress itself, three important determinants of the stress-to-coping balance are investigated: social support (Multidimensional Perceived Social Support Scale), resilience (Resilience Questionnaire), and effort/reward balance (Siegrist Questionnaire).

The 15-item Hungarian version of the Effort-Reward Imbalance (ERI) Questionnaire [90]will be used to measure chronic work-related stress that was reported to predict health conditions [91, 92]. Its core assumption is that the imbalance between high efforts spent and low rewards received in turn leads to an increased risk of poor health. Four indicators can be computed from the raw scores: effort, reward, effort-reward imbalance, and over-commitment. We also aim to screen the participants for burnout (Maschlach Burnout Inventory), an important non-clinical, workplace-based consequence of stress.

In addition to workplace-based psychosocial factors, we plan to collect non-workplace-related interpersonal factors using the following questionnaires. The Shortened Marital Stress Scale (SMSS), a shortened, Hungarian version of the Stockholm Marital Stress Scale [93] will be used to measure the quality of marital relationship [94, 95]. Its validation was performed among patients with cardiovascular diseases and in two studies on the general population in Hungary [96]. The Experiences in Close Relationships-Relationship Structures Questionnaire (SECR-RS) will be used to assess attachment toward the romantic partner [97, 98]. The validated shortened Multidimensional Scale of Perceived Social Support (MSPSS) questionnaire will be used to assess perceptions of social support adequacy from three specific sources: family, friends, and significant others [99, 100].

Sleep is a further important determinant of mental and physical health. Sleep quality and timing (which are important determinants of healthy aging) will be assessed by using a Hungarian adaptation of the Pittsburgh Sleep Quality Index (PSQI-HUN; [101, 102]). The PSQI is a widely used psychometric tool, designed for the evaluation of subjective sleep quality and specific sleep-related symptoms in healthy and clinical populations. Likewise, PSQI-HUN was found to be a valid and reliable measure of sleep quality in Hungarian people [102]. The Hungarian adaptation addresses the difference between work- and free-day sleep timing that are key markers of chronotype and social jetlag [103, 104].

Intelligence is a modifier of psychosocial status and determinant of the response to stress—yet its role was rarely investigated in previous longitudinal studies. Given the time constraints of the study, the International Cognitive Ability Resource (ICAR-16) test will be conducted only in a subsample of the Semmelweis cohort. ICAR-16 [105] is a short, open-source intelligence test that highly correlates with traditional, longer, copyrighted intelligence tests [106]. ICAR-5, an abbreviated version of ICAR-16, will be used in the main Semmelweis Study that is a set of rapidly solvable and variable items that strongly correlates with the results of ICAR-16 [107].

#### Physical health examination and physiological measurements

The clinical screening, consisting of a health assessment by physical examination and physiological measurements, will be performed on the same day as the questionnaire completion. During the screening, we will assess anthropometric parameters, estimate body composition, and measure systolic and diastolic blood pressure by trained research staff according to standard operating protocols. Participants will also take part in a series of specialized physiological examinations focusing on macro- and micro-vascular and cognitive functioning (Table 4).

**Table 4:** Measured parameters and physiological tests at baseline assessment

Blood pressure	
Anthropometric data	Height, weight, waist circumference
Body composition	
Grip strength	
Cognitive function	Multi-domain (attention, psychomotor speed, executive function, spatial working memory, memory)
Peripheral vasculature	Arterial stiffness Microcirculatory reactivity (post-occlusive reactive hyperemia)
Central vasculature	Fundus imaging *Transcranial Doppler sonography
Gait pattern	
Cortical connectivity	*Electroencephalogram (EEG)
	*Functional near-infrared spectroscopy (fNIRS)

^*^These measurements will be performed in sub-groups

Anthropometric parameters (height, sitting height, weight, waist, and hip circumference) will be measured to assess general nutritional and metabolic status. These measures also provide information for derived variables (e.g., body mass index (BMI), waist-to-hip ratio (WHR), waist-to-height ratio, height-to-sitting height ratio). To better characterize body composition, a multi-segment impedance-based measurement is also included [108]. Grip strength (GS) will be measured via a hand dynamometer [109]. Low GS is considered to be a biomarker of sarcopenia [110, 111] and frailty [112].

Blood pressure will be taken in a seated position on the upper arm in duplicates. Ankle-brachial index (ABI) will be measured in the supine position on the upper and lower extremities with a fully automated system. ABI is a strong predictor of macrovascular events and its inclusion was shown to improve the performance and cost-effectiveness of the Framingham Risk Score (FRS) [113, 114].

Carotid-femoral pulse wave velocity (cfPWV), an indicator of arterial stiffness, will be measured by an applanation tonometry-based device. Higher aortic stiffness is a well-accepted independent predictor of first cardiovascular events [115]. Furthermore, using repeat measures of this measure, we can use change in cfPWV (or its trajectory) as an intermediate outcome proving that such “soft” endpoints predict a later occurring disease.

Another important aspect of vascular health is measured as the reactivity of vessels to the changing environment. The inner lining of vessels, the endothelium is a major regulator of vascular diameter, and its preserved function is essential for the normal functioning of blood vessels. The gold standard assessment of vascular reactivity (Flow Mediated Dilation (FMD) test) improved the prognostic value of the FRS [116, 117]; however, it is technically challenging and requires extensive standardization [118]. There are novel methods to assess endothelial function and vascular reactivity (e.g., finger plethysmography, retinal flicker test), but their clinical value requires further longitudinal testing [118]. We will assess microvascular reactivity using laser speckle contrast imaging at the baseline examination of the Semmelweis Study [119].

The participants’ gait will be evaluated in both a control condition and during cognitive stimulation (while counting backwards) using the Protokinetics Gait Analysis Mat. Gait velocity without cognitive stimulation is a recognized indicator of general mobility, with low gait velocity being considered a biomarker of accelerated aging [120] and a predictor of falling, cardiovascular events, and all-cause mortality [121]. In addition to assessing physical condition, gait analysis, particularly during cognitive stimulation, offers valuable insight into the health of the central nervous system. Walking relies on various brain areas, and gait pattern and regulation may change during the development of cognitive impairment or in sub-clinical pathological conditions, such as cerebral small vessel disease [122].

The retina and cerebral microvasculature share similar embryological origins and possess similar structural and functional characteristics (including microvascular barrier function, autoregulation, and neurovascular coupling responses, the role of pericytes and glial cell connections). Thus, retinal microvascular alterations may reflect alterations in the cerebral microvessels (e.g., small vessel disease). Recent studies demonstrate that biological age (see below) and risk for cardiovascular and neurodegenerative diseases can also be predicted on the basis of fundus images [123–125]. We will perform non-mydriatic color fundus photography (FP) on each participant to collect information on different characteristics of the retinal microvasculature (arterial and vein diameter, vascular density, and branching). In addition to frequently used imaging biomarkers, we will search for novel quantitative parameters in collaboration with the ophthalmic reading center of Semmelweis University that could improve the prediction of chronic diseases and aging.

#### Laboratory examinations

Fasting native and anticoagulated blood (serum, plasma, peripheral blood mononuclear cells) and morning first void urine samples will be obtained. Routine bioassays will be performed at the Department of Laboratory Medicine of Semmelweis University (Table 5**)**. Additional samples will be frozen at − 80 °C for later determination with a special emphasis on multi-omic methodologies (genetic paneling, multiprotein arrays, and biomarkers of aging). We will deposit biospecimens in accordance with the Semmelweis University Biobank regulations for future research. These samples will serve several interconnected purposes. First, hypothesis-free testing can be performed to investigate strong predictors of diseases and cause-specific mortality, and their potential to improve disease risk calculators can be assessed. Second, these samples can be involved in large consortia to identify novel predictors and to better characterize their association with different outcomes. Third, novel (frequently not yet known) biomarkers can be measured in stored samples in a case–control or case-cohort design to optimize the use of available resources. For example, proposed biomarkers of aging (e.g., IGF-1 [126, 127]) or cardiovascular disease (e.g., Lp(a) [128]) can be measured in these stored samples.

**Table 5:** Laboratory parameters measured at baseline assessment

Blood samples	
Liver function tests	ALT, ASP, ALP, albumin, total protein, bilirubin, GGT
Kidney function tests	Urea, creatinine, estimated GFR
Pancreatic function test	Lipase assay
Diabetes/insulin resistance screening (+ OGTT)	fasting plasma glucose, HbA1c level, insulin
Fluid and electrolyte panel	Sodium, potassium, calcium, magnesium, chloride, phosphate, bicarbonate
Lipid panel	TC, HDL-C, triglyceride, Apo B
Thyroid	TSH
Inflammation marker	C-reactive protein (hsCRP)
Complete blood count (CBC)	
Other	Uric acid
Urine sample	
Protein panel	Total protein, albumin
Kidney function	Creatinine
Fractionated urinary excretion of Na^+^, K^+^, Ca^2+^	

Abbreviations: *GGT*, gamma-glutamyl transferase; *GOT*, glutamic-oxaloacetic transaminase; *GPT*, glutamic-pyruvic transaminase; *HDL*, high density lipoprotein; *OGTT*, oral glucose tolerance test; *TSH*, thyroid-stimulating hormone; *LDL*, low density lipoprotein; *TC*, total cholesterol; *hsCRP*, high-sensitivity C-reactive protein

Aging is a major risk factor for cardiovascular diseases, and good cardiovascular health is essential to reach advanced age, to avoid disability, and increase the health span of individuals. Hence, a focus of the laboratory measures is to provide parameters for vascular risk estimation, including those used in routine laboratory practice. For example, total cholesterol, HDL-cholesterol, and triglyceride concentrations will be measured to provide a simple estimation of lipid status. In addition, apolipoprotein B (Apo B) will also be measured, as Apo B is thought to be a more accurate marker of cardiovascular risk than LDL-cholesterol or non-HDL-cholesterol [129–131]. Similarly, markers of other organ systems will be routinely measured (hepatic, renal function, blood cell counts, etc.).

Both diabetes mellitus and prediabetes increase the risk of macro- and microvascular disease [132]. While HbA1c-diagnosed diabetes mellitus seems to be sufficient for the diagnosis of diabetes-associated vascular risk from a clinical point of view [133], other markers of glycemia are necessary to better understand the natural history of diabetes development. We have previously shown that different subtypes of diabetes mellitus and prediabetes based on the 75 g oral glucose tolerance test (OGTT) and serum insulin levels have different vascular risks and could improve our understanding of the heterogeneity of type 2 diabetes [134–137]. Furthermore, by describing trajectories of different biomarkers (including glycemic measures), the natural history and the different paths toward diabetes development can be described [52, 134, 138]. Given these considerations, we plan to perform a 3-point OGTT (with fasting, 30-min, 2-h blood draws) to collect samples for the determination of serum glucose and insulin levels.

Serum albumin was formerly considered a biomarker of liver synthetic capacity. However, recent studies have clearly shown that it has several physiologic functions, and it is also a useful biomarker of nutritional status. Furthermore, hypoalbuminemia is associated with poor postoperative outcomes [139, 140]. As the predictive value of albumin in people without an inflammatory status is complex [141], the concurrent measurement of C-reactive protein (CRP) with a hypersensitive assay (hsCRP) in our prospective study allows the investigation of its accuracy as a nutritional biomarker and predictor of different outcomes.

The Semmelweis Study also includes creatinine measurements, GFR calculation, and quantitative urine protein (total and albumin) analysis, which provides a useful insight into early kidney disease, and possibly is a biomarker of microvascular disease [142].

#### Estimation of biological age

The aging process underlies a wide range of chronic diseases (“aging-induced diseases”) and conditions, including cardiovascular and cerebrovascular diseases, neurodegenerative diseases, neoplastic diseases, arthritis, osteoporosis, type 2 diabetes mellitus, and hypertension. This is supported by the fact that chronological age is the most important (yet unmodifiable) risk factor for these diseases. Organismal aging is also characterized by an exponential increase in the age-dependent mortality rate.

The field of geroscience distinguishes between chronological age, which represents the number of years a person has been alive, and biological age, which captures inter-individual differences in age-related molecular, cellular, and functional changes underlying morbidity and mortality. In other words, biological age represents how old a person appears to be, based on his or her physical characteristics (“appearance”), physiological functioning, and molecular, biochemical, and cellular characteristics. Unsuccessful/unhealthy aging is characterized by a biological age that is older than one’s chronological age. People who have biological ages that are older than their chronological ages experience earlier manifestation and higher rates of age-associated diseases and mortality. Successful/healthy aging in contrast is characterized by a biological age that is younger than one’s chronological age. People who have biological ages that are younger than their chronological ages have reduced risk for age-associated diseases and mortality. In the past decade, robust measures of biological age have been developed based on epigenetic markers, biochemical measures, and physical assessments [143–150]. These validated measures of biological age have proven useful for comparing rates of age-related functional decline and predicting age-related outcomes. In the Semmelweis Study, multiple measures of biological age will be calculated to estimate individual rates of aging and to identify sub-groups of people at risk for the development of age-associated chronic diseases [151–155].

In addition to genetic and biochemical parameters, artificial intelligence-assisted and machine-learning techniques are being increasingly used to predict biological age. For example, images of people’s faces [156, 157] can be used to estimate biological age.

In the Semmelweis Study, we plan to collect anonymized images of eye corners or face images as well as fundus photographs of each participant to estimate biological age. Recent studies using deep learning technology can predict retinal microvascular age which was significantly associated with incident cardiovascular diseases as well as mortality and development of neurodegenerative diseases [123–125].

### Logistics of baseline data collection

The baseline data collection will take place on University premises specifically designed for the purposes of the Study. A maximum of 10 people per day are expected during the baseline assessment, one group in the morning and one in the afternoon. Considering the expected participation rate, completion of baseline data collection is anticipated in approximately 3 to 4 years. The personnel dedicated to the Study will be trained to follow standard study procedures.

### Follow-up and expected attrition

Passive data linkage with administrative databases of the National Health Insurance Fund (NHIF) will allow the generation of several study outcomes with dates. The NHIF databases contain information on diagnoses and procedures for out- and inpatient care events, prescriptions filled, sickness benefits, all-cause, and cause-specific mortality for each individual. Furthermore, we will also seek permission to use the National eHealth Infrastructure that contains the electronic health records for all out- and inpatient events including data on imaging and laboratory measurements. While this database has a great opportunity for research use, the different methodologies and provider-related differences require validation before its research use.

In subsequent phases of the Semmelweis Study, we plan to collect similar data to the baseline examination in 5 yearly intervals. This allows comparability of the baseline and subsequent phases and allows adjustment for time-varying covariates that enhance the investigation of independent predictors of different outcomes. Furthermore, we have previously shown that disease prediction may be improved by the use of repeat measures [158]. Repeated data collections allow to use different waves as baselines when developing risk prediction models for a certain time horizon (e.g., 5 or 10 years), thus hugely improving statistical power and facilitating the most parsimonious use of available data [133, 159]. In addition, repeated measures allow us to analyze trajectories of biomarkers that can help in understanding disease development [160–164].

Another potential of repeat data collection in subsequent phases of the Semmelweis Study is to add novel biomarkers and tests that could further enhance our understanding related to the development of a diverse range of age-related pathologies. These biomarkers may yet be unknown or their prohibitive price could preclude their collection at baseline. A non-exhaustive list of these includes the assessment of clonal hematopoiesis of indeterminate potential (CHIP, a common aging-related phenomenon in which mutations in hematopoietic progenitor cells contribute to the clonal proliferation of pro-inflammatory, disease-promoting subpopulation of blood cells), omics-based biomarkers including proteomic biomarkers (e.g., the SomaLogic v4.1 platform allows for the detection and quantification of over 7000 proteins simultaneously in a single sample) and epigenetic clocks (a set of biomarkers that use DNA methylation patterns to estimate a person’s biological age), and the extensive characterization of inflammatory, hormonal and other aging-associated biomarkers (e.g. exosome-based biomarkers). Longitudinal measures of subclinical cardiovascular, cerebrovascular, and neurodegenerative diseases could be gathered through serial ultrasounds and CT or MR imaging. Novel methods of data collection using digital and wearable technologies on lifestyle measures (regarding diet, physical activity, sleep, and social networks) could provide better quality information compared to standard questionnaires. Furthermore, as the cohort is aging, data on hearing and visual impairment, bone and dental health, and pulmonary disease will also be gathered.

To optimize the use of the samples stored in the Semmelweis Study Biobank, we will set up nested case–control and case-cohort studies that are cost-effective ways to investigate novel biomarkers of different outcomes [165].

To cover transitions from a healthy state to the preclinical stage and clinical disease, the length of follow-up is planned for at least 20 years and will depend on the availability of continuous funding.

In addition to investigations covered by the main Semmelweis Study protocol, further embedded studies are also planned that will answer specific questions (such as quality of sleeping, different aspects of aging, or cognitive functioning; pharmacovigilance data [166, 167]) using more detailed phenotyping of the participants using the Semmelweis cohort as the source population. The data collected in the Semmelweis Study will be openly shared with the wider research community in accordance with the FAIR principles [168]. This will likely increase the impact and value of the research, as other researchers can use the data to replicate or build upon the findings, thereby advancing scientific knowledge in the field.

We expect higher response rates at the follow-up examinations compared to the baseline examinations, as was found in other cohort studies [49, 71, 73]. Furthermore, we plan to provide regular feed-back to participants on the findings of the study on the Semmelweis Study webpage and in newsletters. Furthermore, there is a perceived benefit of receiving a free health check at 5-year intervals by highly trained professionals. In addition, a university-wide benefit program is underway in the framework of the “Semmelweis Caring University Program” [42].

### Statistical power calculation

For statistical power [169] calculation, we used the “Episheet” tool, created by Rothman et al. [170] For these calculations, we assumed an overall 70% participation rate leading to an analytical sample of approximately 6200 participants and made the calculation for a 5-year follow-up. Next, we modelled different scenarios based on the cumulative incidence estimated using literature data of the primary outcomes of the Semmelweis Study (metabolic syndrome [171], hypertension [172], diabetes [173], and obesity [174]). For the calculations, we set the alpha level to 5% and statistical power to 80%. We provide a table with the number of participants exposed required for 6 relative risk (RR) levels when the upper tertile (exposed) is compared to the bottom two tertiles for a continuous risk factor (Table 6).

**Table 6:** Sample size calculations for the Semmelweis Study’s primary outcomes and different RR effect levels

Primary outcomes (risk in unexposed)	Relative risk effect levels					
	1.1	1.2	1.3	1.4	1.5	1.6
Type 2 diabetes (0.041)	NA	NA	NA	2095	1466	1048
Hypertension (0.16)	NA	1676	838	629	419	210
Obesity (0.042)	NA	NA	NA	2095	1466	1048
Metabolic syndrome (0.053)	NA	NA	NA	1676	1048	838
Hyperlipidaemia						

NA, not enough statistical power at these parameters

Further, we also provide a table with a number of participants required for hypothetical outcomes with different incidence levels (Table 7). For example, for type 2 diabetes mellitus (after the exclusion of ~ 400 prevalent cases at baseline [175, 176]) as an outcome, with an estimated cumulative incidence of 4.1% in the unexposed group, our study has sufficient power to detect risk factors that have a relative risk of 1.4 or higher (when comparing the top to the bottom two tertiles). For hypertension (that has a higher estimated incidence of 16% and a higher prevalence of 22.6%) [177], the corresponding RR cutoff is 1.2.

**Table 7:** Calculation scenarios based on RR effect levels and incidence in unexposed subjects

Scenarios for 5-year incidence in unexposed (%)	Relative risk-effect levels					
	1.1	1.2	1.3	1.4	1.5	1.6
1	NA	NA	NA	NA	NA	NA
3	NA	NA	NA	NA	1885	1466
5	NA	NA	NA	1676	1048	835
7.5	NA	NA	1886	1048	838	629
10	NA	NA	1467	838	629	419
12.5	NA	NA	1048	629	419	419
15	NA	1886	838	629	419	210

*NA*, not enough statistical power at these parameters

The results of our power calculations are further supported by previous experiences from the Whitehall study that medium-sized prospective cohort studies have sufficient statistical power at similar relative risk levels [158, 159].

## Operational strategies

Although the Semmelweis Study is run using its dedicated team separate from other Semmelweis University–related activities in order to maintain the high quality and confidentiality of the collected data, it is an important part of the overall Semmelweis Caring University Model Program. If a participant is diagnosed with an incident chronic disease based on the laboratory evaluation during Semmelweis Study or is found to have an elevated vascular risk, he/she has the opportunity to visit the Centre of Preventive Services (CPS) of Semmelweis University [42]. The Health Risk Assessment Unit of the CPS provides care for employees with elevated cardiovascular and metabolic risk without manifest chronic disease diagnosis.

The Semmelweis Study is administered by a Semmelweis University-based Management Committee of co-Principal Investigators, supported by a team of national and international consultants, and seeks approval from the Hungarian Medical Research Council and the National Centre for Public Health.

All data collected from questionnaires, physical examination, clinical samples, and data linkage are handled securely and confidentially in accordance with the provisions of the General Data Protection Regulation (GDPR) that was implemented in Hungary and is enforced by the National Authority for Data Protection and Freedom of Information (NAIH). In accordance with the regulations of the GDPR, a data protection impact assessment has been carried out with the Data Protection Center of the University.

All collected information is stored securely in our restricted-access computer network data safe haven (DSH) portal. Administrative data and medical information are stored separately and researchers only have access to pseudonymized records, while the administrative team manages other personal information. The key to combining the 2 databases is only accessible by the study’s senior data manager and the primary investigator through security logging of all access.

## Institutional commitment

Strong institutional commitment and unequivocal support at the leadership level are essential prerequisites for the success of the Semmelweis Study. With this realization, the senior leadership of Semmelweis University positioned the Semmelweis Study as a strategic priority and treated the study as an important component of the university’s ambitious institutional strategy. The university’s commitment to research excellence is well proven by the fact that Semmelweis University has the highest proportion of internationally acclaimed, high-impact, highly cited research of any major Hungarian university. The university’s senior leadership realizes the importance and strategic advantages of establishing a world-class cross-disciplinary research program focusing on the greatest societal challenge that Europe and Hungary face in the upcoming decades. Semmelweis University is committed to facilitating the development of this new research initiative that promotes the development of international collaborations, drives innovation, and supports the development of a national Healthy Aging Program that addresses the vexing and challenging problems of the greying Hungarian society. The University’s leadership uses its institutional grants programs and commits significant development funds provided by the Government of Hungary to establish the infrastructure needed to develop the Semmelweis Study. To promote employee participation, the university’s Department of Human Resources provides a time allowance for participating employees. The university’s Directorate of Legal Affairs assigned a legal counsel to the management team of the Semmelweis Study. The Directorate General of Marketing and Communication is tasked with the promotion of Semmelweis Study within the organization to the faculty, staff, and participating academics. To that end, it maintains contact with the press and creates and edits the web page of the Semmelweis Study, organizes interviews, places articles in the university’s own newspaper, prepares image films, and develops the social media strategy of the Semmelweis Study. As part of this initiative, the Directorate of Brand and Marketing is also tasked with assisting the development of the Semmelweis Study to strengthen the “Semmelweis brand” nationally and internationally and promote a “Semmelweis identity” within the university.

The university’s senior leadership also realizes the importance of diverse multi-investigator and multi-disciplinary research teams, which straddle multiple faculties and departments, working together on focused research problems within the Semmelweis Study using a team approach. The Semmelweis Study advances cross-faculty research collaborations creating productive ties among the research programs of the Faculty of Medicine, Faculty of Health Sciences, and the Faculty of Dentistry. Such ties are expected to reduce the isolation of researchers and enhance their scholarly pursuits, among others. To achieve these goals, the leadership of Semmelweis University offers funding, equipment grants, salary lines, research infrastructure, and encouragement to bring a world-class team of researchers together across disciplines to work on the Semmelweis Study.

## Funding strategy

Funding for the initial infrastructure and the baseline data collection of the study has been provided by a series of grants obtained from the Ministry of Innovation and Technology of Hungary, the National Research, Development and Innovation Fund, the European University for Well-Being (EUniWell) program, and by the leadership of Semmelweis University through the allocation of resources for the purposes of the study. Once the cohort is assembled, continuing support will be sought from Semmelweis University and national and international funding organizations. The leadership team of the Semmelweis Study will apply for research funding from a variety of sponsors, including the National Research, Development and Innovation Fund, the Hungarian Academy of Sciences, the European Research Council, and private foundations. The goal is to secure funding to allow for an increasingly rich data collection in the upcoming phases through personal contact with the cohort participants.

## Main strengths and perspectives

The number of prospective cohort studies ongoing in Hungary is very limited. The cohort ‘18 Growing Up in Hungary [178] is a longitudinal birth cohort initiated in 2018. This countrywide representative study follows 8000 children from before conception and focuses on childbearing and child development in Hungary. Hungary also participates in the Survey of Health, Aging and Retirement in Europe (SHARE) [179], a collaboration that runs population-based, representative waves of panel surveys aimed to give a broad picture of life after the age of 50 in EU countries, including Hungary. We are not aware of ongoing cohort studies in an occupational setting in Hungary or in Eastern Central Europe of similar size and coverage as the Semmelweis Study, especially in a cohort of workers that include healthcare professionals.

We think that our study will be unique and valuable in Hungary and Central Europe for several reasons. The observed associations between predictors and outcomes will help tune disease risk calculators that have either been validated on other populations or relatively long ago. With repeated examinations, the relationship between patient trajectories and disease outcomes can be assessed. Moreover, the effect of environmental and lifestyle factors on certain biological markers can be measured leading to a better understanding of how their effect is mediated in disease outcome and progression.

Based on the experiences of other occupational cohorts, participation in similar cohorts is much higher than in representative samples leading to increased internal validity of the results. Even though the Semmelweis Study is biased by the healthy worker effect and thus not representative of the Hungarian population; based on our observation from the Whitehall II study, the relative risks within the cohort have a good external validity and are close to those observed in population-based samples [180].

The Semmelweis Study will also contribute to the validation of risk prediction models. Risk prediction is crucial in public health and clinical practice to identify high-risk individuals and prevent incident disease through risk-tailored management. Prediction models are also important at the population level to assess the risk of future morbidity and mortality, while at the individual level, risk awareness may motivate people to change their lifestyle or comply with preventive medical advice [181]. There are numerous multivariable models to predict the risk of developing various CVD outcomes in the general population [182]. The most widely used examples are the Framingham risk score (FRS) [183], the SCORE2 [184], QRisk [185], or the population-based Globorisk [186]. The Framingham Health Study has established traditional risk factors for CVD (e.g., age, blood pressure, total cholesterol, smoking status) and was used to develop several risk prediction models for chronic conditions that are included as primary endpoints of the Semmelweis Study. Systematic reviews of risk prediction conclude that studies should focus on validating and tailoring existing risk calculators to local settings and population characteristics. Furthermore, it is suggested that it is better to add potential new predictors to already externally validated models rather than developing new models [182, 187, 188]. In recent years, the integration of omics data (genome, proteome, metabolome) into risk prediction models is becoming popular [189–191]. The clinical success of such models depends on the selection of plausible genetic variants, phenotype reversibility, and effective therapeutic choices based on genotype–phenotype interactions [192–196]. The Semmelweis Study through its broad range of data collection will be able to contribute to better calibrating existing models and drive better policy decisions in Hungary aimed at supporting healthy aging.

An important strength of the Semmelweis Study is its focus on the social determinants of health and disease in a Central European country. We plan to investigate health inequalities and social gradients in unhealthy aging from a social, psychological, and a biomedical perspective. Similar to the Whitehall II study, a potential limitation of the Semmelweis Study is that it will not be representative of employment grades and conditions of other Hungarian workplaces. However, in contrast to the Whitehall II study, it will cover a wider range of socioeconomic and occupational characteristics from manual workers through medical staff working in shifts to administrative and academic persons.. The wide social gradient of employees will lead to a better understanding of the relationship between disease outcome and social status in Hungary. We hypothesize a more rigid occupational hierarchy in a medical school than in other state-run institutions. This can be also construed as a strength of the Semmelweis Study. Occupational hierarchies likely do not vary considerably across other workplaces in higher education; thus, the data obtained in the Semmelweis Study could be potentially compared with other future university-based occupational cohort studies in partnering institutions in the European Union.

The Semmelweis Study will have demographic features that in large reflect the general composition of workforces at other Hungarian universities. Over two-thirds of the employees of Semmelweis University are women, and they are overrepresented in nursing, clerical, and office support jobs. The high proportion of female workers is another important strength. Women are often underrepresented in cohort studies and the reliability of risk calculators in women is often low due to the lower number of women in the included cohorts as well as non-menopausal women’s overall lower cardiovascular morbidity and mortality. Women make up the majority of healthcare workers in the EU (an average of 78%) [197] as well as in Hungary.

Since the cohort includes both healthcare workers and university employees with diverse, not healthcare-related tasks, the health status of healthcare workers can be compared to that of Hungarian office workers. The healthcare industry is a hazardous and stressful workplace environment. Healthcare workers are constantly exposed to a variety of health and safety hazards, including but not limited to exposure to biological risks (e.g., microbial pathogens), toxic chemicals, radiation, noise, sleep deprivation, sleep cycle disruption, and long working hours. There are also a large number of ergonomic issues (including standing for long periods, heavy lifting) and a large variety of psychological stresses (ranging from stress related to heavy patient loads, overly high expectations from patients and superiors, peer pressure, administrative issues, fear of litigation, to financial issues and work-life crossing over into personal life). These issues can lead to a number of serious health problems for healthcare professionals. The Semmelweis Study can potentially provide information on risk factors that can be targeted by specific interventions and could help to preserve the mental and physical health of healthcare workers.

In Europe, there is a series of important longitudinal studies (e.g., the English Longitudinal Study of Ageing [198], the Longitudinal Aging Study Amsterdam [199], the Maastricht Study [200, 201]) following middle-aged samples into aging. The Semmelweis Study will be an important addition to these cohorts as it intends to follow a relatively large sample of employees who are aged 50 and above. Furthermore, the Semmelweis Study is the first comprehensive, multidisciplinary cohort study of aging following people over 50 years in Hungary. The Semmelweis Study, through its repeated data collection over a long-term follow-up period, will enable the investigation of changes in health and ill-health, functional status, health services utilization, and various biological, physical, medical, psychological, social, lifestyle, and economic characteristics of its participants. The results of this study will inform about disease etiology including the long preclinical stages, as well as disease progression, including development of comorbidity and multimorbidity. In addition to the scientific community and healthcare, this information can also be valuable for policymakers, as they can identify important needs and limitations of the current healthcare available for working-age individuals.

The modifiable risk factors investigated in the Semmelweis Study can provide input for the development of complex preventive interventions which can be applied in various workplace-based settings.

### Supplementary information

Below is the link to the electronic supplementary material.

Supplementary file1 (PDF 385 KB)

## References

[1] Eurostat. Ageing Europe. Looking at the lives of older people in the EU. 2020 edition. https://ec.europa.eu/eurostat/documents/3217494/11478057/KS-02-20-655-EN-N.pdf/9b09606c-d4e8-4c33-63d2-3b20d5c19c91?t=1604055531000 (accessed on 05/16/2021).

[2] Eurostat: aging Europe. https://ec.europa.eu/eurostat/cache/digpub/ageing/ (accessed on 11/04/2022).

[3] World Health Organization Regional Office for Europe. Health and well-being and the 2030 Agenda for Sustainable Development in the WHO European Region: an analysis of policy development and implementation. In: Report of the first survey to assess Member States’ activities in relation to the WHO European Region Roadmap to Implement the 2030 Agenda for Sustainable Development. Copenhagen; 2021.

[4] Hungarian Central Statistics Office. STADAT tables 22.1.1.4. https://www.ksh.hu/stadat_files/nep/hu/nep0004.html (accessed on 05/05/2022).

[5] European Commission. The 2015 aging report: underlying assumptions and projection methodologies. Eur Econ. 2014;8. https://ec.europa.eu/economy_finance/publications/european_economy/2014/pdf/ee8_en.pdf. Accessed 05 May 2022.

[6] Ungvari Z, Adany R (2021). The future of healthy aging: translation of geroscience discoveries to public health practice. Eur J Public Health.

[7] Lopez-Otin C, Blasco MA, Partridge L, Serrano M, Kroemer G (2013). The hallmarks of aging. Cell.

[8] Ungvari Z, Tarantini S, Donato AJ, Galvan V, Csiszar A (2018). Mechanisms of vascular aging. Circ Res.

[9] Ungvari Z, Tarantini S, Sorond F, Merkely B, Csiszar A (2020). Mechanisms of vascular aging, a geroscience perspective: JACC focus seminar. J Am Coll Cardiol.

[10] Rowe JW, Kahn RL (1987). Human aging: usual and successful. Science.

[11] Rowe JW, Kahn RL (1997). Successful aging. Gerontologist.

[12] Rowe JW, Kahn RL (2015). Successful aging 2.0: conceptual expansions for the 21st century. J Gerontol B Psychol Sci Soc Sci..

[13] World Health Organization. Decade of healthy ageing: baseline report. 2020. https://iris.who.int/handle/10665/338677.

[14] Choi H, Steptoe A, Heisler M, Clarke P, Schoeni RF, Jivraj S, Cho TC, Langa KM (2020). Comparison of health outcomes among high- and low-income adults aged 55 to 64 years in the US vs England. JAMA Intern Med.

[15] OECD/European observatory on health systems and policies. State of health in the EU. Hungary: Country Health Profile; 2021. 10.1787/482f3633-en.

[16] Average life expectancy at birth. Hungarian Central Statistical Office. https://www.ksh.hu/stadat_files/nep/hu/nep0060.html (accessed on 09/01/2022).

[17] Eurostat. Aging Europe - Looking at the lives of older people in the EU - 2020 ed. 2020. 10.2785/628105.

[18] Schöley J, Aburto JM, Kashnitsky I, Kniffka MS, Zhang L, Jaadla H, Dowd JB, Kashyap R (2022). Life expectancy changes since COVID-19. Nat Hum Behav..

[19] OECD/European Union. Health at a Glance: Europe 2020: State of health in the EU cycle. State of Health in the EU Cycle; 2020. 10.1787/82129230-en.

[20] OECD. Avoidable mortality (preventable and treatable). In: Health at a Glance 2021: OECD Indicators. Paris: OECD Publishing; 2021. 10.1787/ae3016b9-en.

[21] Global Burden of Disease Collaborative Network. Global Burden of Disease Study 2019 (GBD 2019) Reference Life Table. Seattle, United States of America: Institute for Health Metrics and Evaluation (IHME); 2021. 10.6069/1D4Y-YQ37

[22] Hungarian Central Statistics Office. Healthy life years 4.1.1.41. https://www.ksh.hu/stadat_files/ege/en/ege0041.html?msclkid=564b2b7bcf7111ecbb974e8589019613 (accessed on 05/09/2022).

[23] OECD/European Observatory on Health Systems and Policies. Hungary: Country Health Profile 2019. State of Health in the EU, OECD Publishing, Paris/European Observatory on Health Systems and Policies, Brussels. 2019. 10.1787/4b7ba48c-en.

[24] Diseases GBD, Injuries C (2020). Global burden of 369 diseases and injuries in 204 countries and territories, 1990–2019: a systematic analysis for the Global Burden of Disease Study 2019. Lancet.

[25] Péterfi A, Mészáros Á, Szarvas Z, Pénzes M, Fekete M, Fehér Á, Lehoczki A, Csípő T, Fazekas-Pongor V. Comorbidities and increased mortality of COVID-19 among the elderly: A systematic review. Physiol Int. 2022. 10.1556/2060.2022.00206. 10.1556/2060.2022.00206 35575986

[26] Nikolich-Zugich J, Knox KS, Rios CT, Natt B, Bhattacharya D, Fain MJ (2020). SARS-CoV-2 and COVID-19 in older adults: what we may expect regarding pathogenesis, immune responses, and outcomes. Geroscience.

[27] Oroszi B, Juhász A, Nagy C, Horváth JK, McKee M, Ádány R. Unequal burden of COVID-19 in Hungary: a geographical and socioeconomic analysis of the second wave of the pandemic. BMJ Glob Health. 2021;6(9):e006427. 10.1136/bmjgh-2021-006427. 10.1136/bmjgh-2021-006427 PMC8438581 34518205

[28] Oroszi B, Juhász A, Nagy C, Horváth JK, Komlós KE, Túri G, McKee M, Ádány R (2022). Characteristics of the third COVID-19 pandemic wave with special focus on socioeconomic inequalities in morbidity, mortality and the uptake of COVID-19 vaccination in Hungary. J Pers Med..

[29] Fast JE, Williamson DL, Keating NC (1999). The hidden costs of informal elder care. J Fam Econ Issues.

[30] Aiyar S, Ebeke CH, Shao X. The impact of workforce aging on European productivity. Int Monet Fund (IMF). 2016:No. 16/238. European Department, IMF Working Paper.

[31] Ahtonen A. Healthy and active ageing: turning the ‘silver’ economy into gold. Europ Policy Cent Policy Brief; 2012.

[32] Masters R, Anwar E, Collins B, Cookson R, Capewell S (2017). Return on investment of public health interventions: a systematic review. J Epidemiol Community Health.

[33] Huszár Á. Osztályszerkezet és jövedelemeloszlás Magyarországon 1982 és 2019 között [Class structure and income distribution in Hungary between 1982 and 2019]. TÁRKI: Társadalmi Riport; 2022.

[34] Uzzoli A (2016). Health inequalities regarding territorial differences in Hungary by discussing life expectancy. Regional Statistics.

[35] Balasubramanian P, Kiss T, Tarantini S, Nyul-Toth A, Ahire C, Yabluchanskiy A, Csipo T, Lipecz A, Tabak A, Institoris A, Csiszar A, Ungvari Z (2021). Obesity-induced cognitive impairment in older adults: a microvascular perspective. Am J Physiol Heart Circ Physiol.

[36] Horvath S, Erhart W, Brosch M, Ammerpohl O, von Schonfels W, Ahrens M, Heits N, Bell JT, Tsai PC, Spector TD, Deloukas P, Siebert R, Sipos B, Becker T, Rocken C, Schafmayer C, Hampe J (2014). Obesity accelerates epigenetic aging of human liver. Proc Natl Acad Sci U S A.

[37] Tucsek Z, Toth P, Sosnowska D, Gautam T, Mitschelen M, Koller A, Szalai G, Sonntag WE, Ungvari Z, Csiszar A (2014). Obesity in aging exacerbates blood-brain barrier disruption, neuroinflammation, and oxidative stress in the mouse hippocampus: effects on expression of genes involved in beta-amyloid generation and Alzheimer's disease. J Gerontol A Biol Sci Med Sci.

[38] Kivimaki M, Strandberg T, Pentti J, Nyberg ST, Frank P, Jokela M, Ervasti J, Suominen SB, Vahtera J, Sipila PN, Lindbohm JV, Ferrie JE (2022). Body-mass index and risk of obesity-related complex multimorbidity: an observational multicohort study. Lancet Diabetes Endocrinol.

[39] Bagyura Z, Kiss L, Lux A, Csobay-Novak C, Jermendy AL, Polgar L, Szelid Z, Soos P, Merkely B (2020). Association between coronary atherosclerosis and visceral adiposity index. Nutr Metab Cardiovasc Dis.

[40] Martos T, Csabai M, Bagyura Z, Ocsovszky Z, Rafael B, Sallay V, Merkely B (2020). Cardiovascular disease risk perception in a Hungarian community sample: psychometric evaluation of the ABCD Risk Perception Questionnaire. BMJ Open.

[41] Merkely B, Szabo AJ, Kosztin A, Berenyi E, Sebestyen A, Lengyel C, Merkely G, Karady J, Varkonyi I, Papp C, Miseta A, Betlehem J, Burian K, Csoka I, Vasarhelyi B, Ludwig E, Prinz G, Sinko J, Hanko B, Varga P, Fulop GA, Mag K, Voko Z; Investigators HUC-ER. Novel coronavirus epidemic in the Hungarian population, a cross-sectional nationwide survey to support the exit policy in Hungary. Geroscience. 2020;42:1063–1074. 10.1007/s11357-020-00226-9 PMC7366154 32677025

[42] Ungvari Z, Adany R, Szabo AJ, Dornyei G, Moizs M, Purebl G, Kalabay L, Varga P, Torzsa P, Kellermayer M, Merkely B (2021). Semmelweis Caring University Model Program based on the development of a center of preventive services: health for all employees at a university occupational setting. Front Public Health.

[43] Doll R, Hill AB (1954). The mortality of doctors in relation to their smoking habits; a preliminary report. Br Med J.

[44] Doll R, Peto R, Boreham J, Sutherland I (2004). Mortality in relation to smoking: 50 years’ observations on male British doctors. BMJ.

[45] Nurses’ Health Study official site. https://nurseshealthstudy.org/?page_id=73 accessed on 09/04/2022.

[46] Checkoway H, Eisen EA (1998). Developments in occupational cohort studies. Epidemiol Rev.

[47] Knutsson A, Akerstedt T, Jonsson BG, Orth-Gomer K (1986). Increased risk of ischaemic heart disease in shift workers. Lancet.

[48] Martikainen P, Lahelma E, Marmot M, Sekine M, Nishi N, Kagamimori S (2004). A comparison of socioeconomic differences in physical functioning and perceived health among male and female employees in Britain, Finland and Japan. Soc Sci Med.

[49] Marmot M, Brunner E (2005). Cohort Profile: the Whitehall II study. Int J Epidemiol.

[50] Kuper H, Marmot M (2003). Job strain, job demands, decision latitude, and risk of coronary heart disease within the Whitehall II study. J Epidemiol Community Health.

[51] Kivimaki M, Nyberg ST, Batty GD, Fransson EI, Heikkila K, Alfredsson L, Bjorner JB, Borritz M, Burr H, Casini A, Clays E, De Bacquer D, Dragano N, Ferrie JE, Geuskens GA, Goldberg M, Hamer M, Hooftman WE, Houtman IL, Joensuu M, Jokela M, Kittel F, Knutsson A, Koskenvuo M, Koskinen A, Kouvonen A, Kumari M, Madsen IE, Marmot MG, Nielsen ML, Nordin M, Oksanen T, Pentti J, Rugulies R, Salo P, Siegrist J, Singh-Manoux A, Suominen SB, Vaananen A, Vahtera J, Virtanen M, Westerholm PJ, Westerlund H, Zins M, Steptoe A, Theorell T; Consortium IP-W. Job strain as a risk factor for coronary heart disease: a collaborative meta-analysis of individual participant data. Lancet. 2012;380:1491–7. 10.1016/S0140-6736(12)60994-5 PMC3486012 22981903

[52] Tabak AG, Jokela M, Akbaraly TN, Brunner EJ, Kivimaki M, Witte DR (2009). Trajectories of glycaemia, insulin sensitivity, and insulin secretion before diagnosis of type 2 diabetes: an analysis from the Whitehall II study. Lancet.

[53] Bell JA, Hamer M, Sabia S, Singh-Manoux A, Batty GD, Kivimaki M (2015). The natural course of healthy obesity over 20 years. J Am Coll Cardiol.

[54] Topiwala A, Allan CL, Valkanova V, Zsoldos E, Filippini N, Sexton C, Mahmood A, Fooks P, Singh-Manoux A, Mackay CE, Kivimaki M, Ebmeier KP (2017). Moderate alcohol consumption as risk factor for adverse brain outcomes and cognitive decline: longitudinal cohort study. BMJ.

[55] Visseren FLJ, Mach F, Smulders YM, Carballo D, Koskinas KC, Back M, Benetos A, Biffi A, Boavida JM, Capodanno D, Cosyns B, Crawford C, Davos CH, Desormais I, Di Angelantonio E, Franco OH, Halvorsen S, Hobbs FDR, Hollander M, Jankowska EA, Michal M, Sacco S, Sattar N, Tokgozoglu L, Tonstad S, Tsioufis KP, van Dis I, van Gelder IC, Wanner C, Williams B; Societies ESCNC and Group ESCSD. 2021 ESC Guidelines on cardiovascular disease prevention in clinical practice. Eur Heart J. 2021;42:3227-3337

[56] Livingston G, Huntley J, Sommerlad A, Ames D, Ballard C, Banerjee S, Brayne C, Burns A, Cohen-Mansfield J, Cooper C, Costafreda SG, Dias A, Fox N, Gitlin LN, Howard R, Kales HC, Kivimaki M, Larson EB, Ogunniyi A, Orgeta V, Ritchie K, Rockwood K, Sampson EL, Samus Q, Schneider LS, Selbaek G, Teri L, Mukadam N (2020). Dementia prevention, intervention, and care: 2020 report of the Lancet Commission. Lancet.

[57] Descatha A, Sembajwe G, Pega F, Ujita Y, Baer M, Boccuni F, Di Tecco C, Duret C, Evanoff BA, Gagliardi D, Godderis L, Kang SK, Kim BJ, Li J, Magnusson Hanson LL, Marinaccio A, Ozguler A, Pachito D, Pell J, Pico F, Ronchetti M, Roquelaure Y, Rugulies R, Schouteden M, Siegrist J, Tsutsumi A, Iavicoli S (2020). The effect of exposure to long working hours on stroke: a systematic review and meta-analysis from the WHO/ILO Joint Estimates of the Work-related Burden of Disease and Injury. Environ Int.

[58] Ferrucci L, Gonzalez-Freire M, Fabbri E, Simonsick E, Tanaka T, Moore Z, Salimi S, Sierra F, de Cabo R (2020). Measuring biological aging in humans: a quest. Aging Cell.

[59] Marmot MG, Smith GD, Stansfeld S, Patel C, North F, Head J, White I, Brunner E, Feeney A (1991). Health inequalities among British civil servants: the Whitehall II study. Lancet.

[60] Ferrie JE, Shipley MJ, Davey Smith G, Stansfeld SA, Marmot MG (2002). Change in health inequalities among British civil servants: the Whitehall II study. J Epidemiol Community Health.

[61] Marmot MG, Shipley MJ, Hemingway H, Head J, Brunner EJ (2008). Biological and behavioural explanations of social inequalities in coronary heart disease: the Whitehall II study. Diabetologia.

[62] Brunner EJ, Shipley MJ, Ahmadi-Abhari S, Valencia Hernandez C, Abell JG, Singh-Manoux A, Kawachi I, Kivimaki M (2018). Midlife contributors to socioeconomic differences in frailty during later life: a prospective cohort study. Lancet Public Health.

[63] Tanaka A, Shipley MJ, Welch CA, Groce NE, Marmot MG, Kivimaki M, Singh-Manoux A, Brunner EJ (2018). Socioeconomic inequality in recovery from poor physical and mental health in mid-life and early old age: prospective Whitehall II cohort study. J Epidemiol Community Health.

[64] Stringhini S, Dugravot A, Shipley M, Goldberg M, Zins M, Kivimaki M, Marmot M, Sabia S, Singh-Manoux A (2011). Health behaviours, socioeconomic status, and mortality: further analyses of the British Whitehall II and the French GAZEL prospective cohorts. PLoS Med.

[65] Virtanen M, Ferrie JE, Tabak AG, Akbaraly TN, Vahtera J, Singh-Manoux A, Kivimaki M (2014). Psychological distress and incidence of type 2 diabetes in high-risk and low-risk populations: the Whitehall II Cohort Study. Diabetes Care.

[66] Stringhini S, Batty GD, Bovet P, Shipley MJ, Marmot MG, Kumari M, Tabak AG, Kivimaki M (2013). Association of lifecourse socioeconomic status with chronic inflammation and type 2 diabetes risk: the Whitehall II prospective cohort study. PLoS Med.

[67] Stringhini S, Tabak AG, Akbaraly TN, Sabia S, Shipley MJ, Marmot MG, Brunner EJ, Batty GD, Bovet P, Kivimaki M (2012). Contribution of modifiable risk factors to social inequalities in type 2 diabetes: prospective Whitehall II cohort study. BMJ.

[68] Okanagan Charter: an international charter for health promoting universities and colleges. 2015. available at https://open.library.ubc.ca/cIRcle/collections/53926/items/1.0132754 (accessesd on 04/17/2021).

[69] WHO's work on the UN decade of healthy ageing (2021–2030). https://www.who.int/initiatives/decade-of-healthy-ageing (accessed on 03/15/2023).

[70] Goldberg M, Leclerc A, Bonenfant S, Chastang JF, Schmaus A, Kaniewski N, Zins M (2007). Cohort profile: the GAZEL cohort study. Int J Epidemiol.

[71] Lahelma E, Aittomaki A, Laaksonen M, Lallukka T, Martikainen P, Piha K, Rahkonen O, Saastamoinen P (2013). Cohort profile: the Helsinki Health Study. Int J Epidemiol.

[72] Kouvonen A, Kivimaki M, Virtanen M, Pentti J, Vahtera J (2005). Work stress, smoking status, and smoking intensity: an observational study of 46,190 employees. J Epidemiol Community Health.

[73] Hvidtfeldt UA, Bjorner JB, Jensen JH, Breinegaard N, Hasle P, Bonde JPE, Rod NH (2017). Cohort profile: the well-being in hospital employees (WHALE) study. Int J Epidemiol.

[74] Hungarian Central Statistical Office. European Health Interview Survey, 2014. Statistical Mirror. 2015/29. 2015. https://www.ksh.hu/docs/hun/xftp/stattukor/elef14.pdf.

[75] Engler Á, Purebl G, Susánszky É, Székely A. Magyar Lelkiállapot 2021: Család- egészség - közösség. Hungarostudy 2021 Tanulmányok [Hungarian State of Mind 2021: Family - Health - Community. Hungarostudy 2021 Studies]. Kopp Mária Intézet a Népesedésért és a Családokért, Budapest; 2022.

[76] Eurostat. European Health Interview Survey (EHIS wave 2) methodological manual. Luxembourg: Publications Office of the European Union, European Commission Eurostat; 2013.

[77] Heikkila K, Nyberg ST, Theorell T, Fransson EI, Alfredsson L, Bjorner JB, Bonenfant S, Borritz M, Bouillon K, Burr H, Dragano N, Geuskens GA, Goldberg M, Hamer M, Hooftman WE, Houtman IL, Joensuu M, Knutsson A, Koskenvuo M, Koskinen A, Kouvonen A, Madsen IE, Magnusson Hanson LL, Marmot MG, Nielsen ML, Nordin M, Oksanen T, Pentti J, Salo P, Rugulies R, Steptoe A, Suominen S, Vahtera J, Virtanen M, Vaananen A, Westerholm P, Westerlund H, Zins M, Ferrie JE, Singh-Manoux A, Batty GD, Kivimaki M; Consortium IP-W. Work stress and risk of cancer: meta-analysis of 5700 incident cancer events in 116,000 European men and women. BMJ. 2013;346:f165. 10.1136/bmj.f165 PMC3567204 23393080

[78] Kivimaki M, Pentti J, Ferrie JE, Batty GD, Nyberg ST, Jokela M, Virtanen M, Alfredsson L, Dragano N, Fransson EI, Goldberg M, Knutsson A, Koskenvuo M, Koskinen A, Kouvonen A, Luukkonen R, Oksanen T, Rugulies R, Siegrist J, Singh-Manoux A, Suominen S, Theorell T, Vaananen A, Vahtera J, Westerholm PJM, Westerlund H, Zins M, Strandberg T, Steptoe A, Deanfield J; Consortium IP-W. Work stress and risk of death in men and women with and without cardiometabolic disease: a multicohort study. Lancet Diabetes Endocrinol. 2018;6:705–713. 10.1016/S2213-8587(18)30140-2 PMC6105619 29884468

[79] Kivimaki M, Virtanen M, Elovainio M, Kouvonen A, Vaananen A, Vahtera J (2006). Work stress in the etiology of coronary heart disease–a meta-analysis. Scand J Work Environ Health.

[80] Kouvonen A, Kivimaki M, Cox SJ, Cox T, Vahtera J (2005). Relationship between work stress and body mass index among 45,810 female and male employees. Psychosom Med.

[81] Siegrist J, Li J (2017). Work stress and altered biomarkers: a synthesis of findings based on the effort-reward imbalance model. Int J Environ Res Public Health..

[82] Levi L, Bartley M, Marmot M, Karasek R, Theorell T, Siegrist J, Peter R, Belkic K, Savic C, Schnall P, Landsbergis P (2000). Stressors at the workplace: theoretical models. Occup Med.

[83] Alfredsson L, Karasek R, Theorell T (1982). Myocardial infarction risk and psychosocial work environment: an analysis of the male Swedish working force. Soc Sci Med.

[84] Karasek R, Baker D, Marxer F, Ahlbom A, Theorell T (1981). Job decision latitude, job demands, and cardiovascular disease: a prospective study of Swedish men. Am J Public Health.

[85] Karasek RA, Theorell T, Schwartz JE, Schnall PL, Pieper CF, Michela JL (1988). Job characteristics in relation to the prevalence of myocardial infarction in the US Health Examination Survey (HES) and the Health and Nutrition Examination Survey (HANES). Am J Public Health.

[86] Karasek RA, Theorell TG, Schwartz J, Pieper C, Alfredsson L (1982). Job, psychological factors and coronary heart disease. Swedish prospective findings and US prevalence findings using a new occupational inference method. Adv Cardiol..

[87] Theorell T, Karasek RA (1996). Current issues relating to psychosocial job strain and cardiovascular disease research. J Occup Health Psychol.

[88] Stauder A, Konkolÿ-Thege B (2006). Az észlelt stressz kérdőív (PSS) magyar verziójának jellemzői. Mentálhigiéné és Pszichoszomatika.

[89] Cohen S, Kamarck T, Mermelstein R (1983). A global measure of perceived stress. J Health Soc Behav.

[90] Salavecz GN, Neculai K, Rózsa S, Kopp M (2006). Az Erőfeszítés-Jutalom Egyensúlytalanság Kérdőív magyar változatának megbízhatósága és érvényessége. Mentálhigiéné és Pszichoszomatika..

[91] Siegrist J, Starke D, Chandola T, Godin I, Marmot M, Niedhammer I, Peter R (2004). The measurement of effort-reward imbalance at work: European comparisons. Soc Sci Med.

[92] Dragano N, Siegrist J, Nyberg ST, Lunau T, Fransson EI, Alfredsson L, Bjorner JB, Borritz M, Burr H, Erbel R, Fahlen G, Goldberg M, Hamer M, Heikkila K, Jockel KH, Knutsson A, Madsen IEH, Nielsen ML, Nordin M, Oksanen T, Pejtersen JH, Pentti J, Rugulies R, Salo P, Schupp J, Singh-Manoux A, Steptoe A, Theorell T, Vahtera J, Westerholm PJM, Westerlund H, Virtanen M, Zins M, Batty GD, Kivimaki M; Consortium IP-W. Effort-reward imbalance at work and incident coronary heart disease: a multicohort study of 90,164 individuals. Epidemiology. 2017;28:619–626. 10.1097/EDE.0000000000000666 PMC5457838 28570388

[93] Orth-Gomer K, Wamala SP, Horsten M, Schenck-Gustafsson K, Schneiderman N, Mittleman MA (2000). Marital stress worsens prognosis in women with coronary heart disease: the Stockholm Female Coronary Risk Study. JAMA.

[94] Balog P, Szekely A, Szabo G, Kopp M (2006). A röviditett házastarsi stressz skála pszichometriai jellemzői. Mentálhigiéné és Pszichoszomatika.

[95] Balog P, Susánszky A (2022). A házastársi/élettársi kapcsolat minősége és a mentális egészségi állapot összefüggései a fiatal felnőttek körében. KAPOCS.

[96] Kopp MS, Thege BK, Balog P, Stauder A, Salavecz G, Rozsa S, Purebl G, Adam S (2010). Measures of stress in epidemiological research. J Psychosom Res.

[97] Jantek G, Vargha A (2016). A felnőtt kötődés korszerű mérési lehetősége: A közvetlen kapcsolatok élményei — kapcsolati struktúrák (ECR-RS) kötődési kérdőív magyar adaptációja párkapcsolatban élő felnőtt személyeknél. Magyar Pszichológiai Szemle.

[98] Fraley RC, Heffernan ME, Vicary AM, Brumbaugh CC (2011). The experiences in close relationships-relationship structures questionnaire: a method for assessing attachment orientations across relationships. Psychol Assess.

[99] Zimet GD, Dahlem NW, Zimet SG, Farley GK (1988). The multidimensional scale of perceived social support. J Pers Assess.

[100] Papp-Zipernovszky O, Kékesi MZ, Kékesi MZ, Jámbori S (2017). A Multidimenzionális észlelt társastámogatás kérdőív magyar nyelvű validálása. Mentálhigiéné és Pszichoszomatika..

[101] Buysse DJ, Reynolds CF, Monk TH, Berman SR, Kupfer DJ (1989). The Pittsburgh Sleep Quality Index: a new instrument for psychiatric practice and research. Psychiatry Res.

[102] Takacs J, Bodizs R, Ujma PP, Horvath K, Rajna P, Harmat L (2016). Reliability and validity of the Hungarian version of the Pittsburgh Sleep Quality Index (PSQI-HUN): comparing psychiatric patients with control subjects. Sleep Breath.

[103] Roenneberg T (2012). What is chronotype?. Sleep Biol Rhythms.

[104] Roenneberg T, Pilz LK, Zerbini G, Winnebeck EC (2019). Chronotype and social jetlag: A (self-) critical review. Biology (Basel)..

[105] Dworak EM, Revelle W, Doebler P, Condon DM (2021). Using the International Cognitive Ability Resource as an open source tool to explore individual differences in cognitive ability. Personality Individ Differ.

[106] Young SR, Keith TZ (2020). An examination of the convergent validity of the ICAR16 and WAIS-IV. J Psychoeduc Assess.

[107] Kirkegaard EOW, Bjerrekær JD. ICAR5: design and validation of a 5-item public domain cognitive ability test. Open Differ Psychol. 2016. 10.26775/odp.2016.07.11.

[108] Ogawa H, Fujitani K, Tsujinaka T, Imanishi K, Shirakata H, Kantani A, Hirao M, Kurokawa Y, Utsumi S (2011). InBody 720 as a new method of evaluating visceral obesity. Hepatogastroenterology.

[109] Bohannon RW (2019). Grip strength: an indispensable biomarker for older adults. Clin Interv Aging.

[110] Kitamura A, Seino S, Abe T, Nofuji Y, Yokoyama Y, Amano H, Nishi M, Taniguchi Y, Narita M, Fujiwara Y, Shinkai S (2021). Sarcopenia: prevalence, associated factors, and the risk of mortality and disability in Japanese older adults. J Cachexia Sarcopenia Muscle.

[111] Bahat G, Tufan A, Tufan F, Kilic C, Akpinar TS, Kose M, Erten N, Karan MA, Cruz-Jentoft AJ (2016). Cut-off points to identify sarcopenia according to European Working Group on Sarcopenia in Older People (EWGSOP) definition. Clin Nutr.

[112] Syddall H, Cooper C, Martin F, Briggs R, Aihie SA (2003). Is grip strength a useful single marker of frailty?. Age Ageing.

[113] Collaboration ABI (2008). Ankle brachial index combined with Framingham Risk Score to predict cardiovascular events and mortality: a meta-analysis. JAMA.

[114] Cortesi PA, Maloberti A, Micale M, Pagliarin F, Antonazzo IC, Mazzaglia G, Giannattasio C, Mantovani LG (2021). Costs and effects of cardiovascular risk reclassification using the ankle-brachial index (ABI) in addition to the Framingham risk scoring in women. Atherosclerosis.

[115] Mitchell GF, Hwang SJ, Vasan RS, Larson MG, Pencina MJ, Hamburg NM, Vita JA, Levy D, Benjamin EJ (2010). Arterial stiffness and cardiovascular events: the Framingham Heart Study. Circulation.

[116] Rossi R, Nuzzo A, Origliani G, Modena MG (2008). Prognostic role of flow-mediated dilation and cardiac risk factors in post-menopausal women. J Am Coll Cardiol.

[117] Yeboah J, Folsom AR, Burke GL, Johnson C, Polak JF, Post W, Lima JA, Crouse JR, Herrington DM (2009). Predictive value of brachial flow-mediated dilation for incident cardiovascular events in a population-based study: the multi-ethnic study of atherosclerosis. Circulation.

[118] Alexander Y, Osto E, Schmidt-Trucksass A, Shechter M, Trifunovic D, Duncker DJ, Aboyans V, Back M, Badimon L, Cosentino F, De Carlo M, Dorobantu M, Harrison DG, Guzik TJ, Hoefer I, Morris PD, Norata GD, Suades R, Taddei S, Vilahur G, Waltenberger J, Weber C, Wilkinson F, Bochaton-Piallat ML, Evans PC (2021). Endothelial function in cardiovascular medicine: a consensus paper of the European Society of Cardiology Working Groups on Atherosclerosis and Vascular Biology, Aorta and Peripheral Vascular Diseases, Coronary Pathophysiology and Microcirculation, and Thrombosis. Cardiovasc Res.

[119] Csipo T, Lipecz A, Fulop GA, Hand RA, Ngo BN, Dzialendzik M, Tarantini S, Balasubramanian P, Kiss T, Yabluchanska V, Silva-Palacios F, Courtney DL, Dasari TW, Sorond F, Sonntag WE, Csiszar A, Ungvari Z, Yabluchanskiy A (2019). Age-related decline in peripheral vascular health predicts cognitive impairment. Geroscience.

[120] Binotto MA, Lenardt MH, Rodriguez-Martinez MDC (2018). Physical frailty and gait speed in community elderly: a systematic review. Rev Esc Enferm USP.

[121] Newman AB, Simonsick EM, Naydeck BL, Boudreau RM, Kritchevsky SB, Nevitt MC, Pahor M, Satterfield S, Brach JS, Studenski SA, Harris TB (2006). Association of long-distance corridor walk performance with mortality, cardiovascular disease, mobility limitation, and disability. JAMA.

[122] Tian Q, Chastan N, Bair WN, Resnick SM, Ferrucci L, Studenski SA (2017). The brain map of gait variability in aging, cognitive impairment and dementia-a systematic review. Neurosci Biobehav Rev.

[123] Hu W, Wang W, Wang Y, et al. Retinal age gap as a predictive biomarker of future risk of Parkinson's disease. Age and Ageing. 2022;51(3):afac062. 10.1093/ageing/afac062. 10.1093/ageing/afac062 PMC8966015 35352798

[124] Zhu Z, Chen Y, Wang W, Wang Y, Hu W, Shang X, Liao H, Shi D, Huang Y, Ha J, Tan Z, Kiburg KV, Zhang X, Tang S, Yu H, Yang X, He M (2022). Association of retinal age gap with arterial stiffness and incident cardiovascular disease. Stroke..

[125] Zhu Z, Shi D, Guankai P, Tan Z, Shang X, Hu W, Liao H, Zhang X, Huang Y, Yu H, Meng W, Wang W, Ge Z, Yang X, He M (2023). Retinal age gap as a predictive biomarker for mortality risk. Br J Ophthalmol..

[126] Ungvari Z, Csiszar A (2012). The emerging role of IGF-1 deficiency in cardiovascular aging: recent advances. J Gerontol A Biol Sci Med Sci.

[127] Sonntag WE, Deak F, Ashpole N, Toth P, Csiszar A, Freeman W, Ungvari Z (2013). Insulin-like growth factor-1 in CNS and cerebrovascular aging. Front Aging Neurosci.

[128] Miksenas H, Januzzi JL, Natarajan P (2021). Lipoprotein(a) and cardiovascular diseases. JAMA.

[129] Grundy SM, Stone NJ, Bailey AL, Beam C, Birtcher KK, Blumenthal RS, Braun LT, de Ferranti S, Faiella-Tommasino J, Forman DE, Goldberg R, Heidenreich PA, Hlatky MA, Jones DW, Lloyd-Jones D, Lopez-Pajares N, Ndumele CE, Orringer CE, Peralta CA, Saseen JJ, Smith SC, Sperling L, Virani SS, Yeboah J (2019). 2018 AHA/ACC/AACVPR/AAPA/ABC/ACPM/ADA/AGS/APhA/ASPC/NLA/PCNA guideline on the management of blood cholesterol: a report of the American College of Cardiology/American Heart Association Task Force on clinical practice guidelines. Circulation.

[130] Sniderman AD, Robinson JG (2019). ApoB in clinical care: pro and con. Atherosclerosis.

[131] Sniderman AD, Pencina M, Thanassoulis G (2019). ApoB. Circ Res.

[132] Tabak AG, Herder C, Rathmann W, Brunner EJ, Kivimaki M (2012). Prediabetes: a high-risk state for diabetes development. Lancet.

[133] Tabak AG, Brunner EJ, Lindbohm JV, Singh-Manoux A, Shipley MJ, Sattar N, Kivimaki M (2022). Risk of macrovascular and microvascular disease in diabetes diagnosed using oral glucose tolerance test with and without confirmation by hemoglobin A1c: the Whitehall II cohort study. Circulation.

[134] Faerch K, Witte DR, Brunner EJ, Kivimaki M, Tabak A, Jorgensen ME, Ekelund U, Vistisen D (2017). Physical activity and improvement of glycemia in prediabetes by different diagnostic criteria. J Clin Endocrinol Metab.

[135] Vistisen D, Kivimaki M, Perreault L, Hulman A, Witte DR, Brunner EJ, Tabak A, Jorgensen ME, Faerch K (2019). Reversion from prediabetes to normoglycaemia and risk of cardiovascular disease and mortality: the Whitehall II cohort study. Diabetologia.

[136] Vistisen D, Witte DR, Brunner EJ, Kivimaki M, Tabak A, Jorgensen ME, Faerch K (2018). Risk of cardiovascular disease and death in individuals with prediabetes defined by different criteria: the Whitehall II study. Diabetes Care.

[137] Wagner R, Heni M, Tabak AG, Machann J, Schick F, Randrianarisoa E, de Angelis HM, Birkenfeld AL, Stefan N, Peter A, Haring HU, Fritsche A (2021). Pathophysiology-based subphenotyping of individuals at elevated risk for type 2 diabetes. Nat Med..

[138] Vistisen D, Witte DR, Tabak AG, Herder C, Brunner EJ, Kivimaki M, Faerch K (2014). Patterns of obesity development before the diagnosis of type 2 diabetes: the Whitehall II cohort study. PLoS Med.

[139] Kim S, McClave SA, Martindale RG, Miller KR, Hurt RT (2017). Hypoalbuminemia and clinical outcomes: what is the mechanism behind the relationship?. Am Surg.

[140] van Stijn MF, Korkic-Halilovic I, Bakker MS, van der Ploeg T, van Leeuwen PA, Houdijk AP (2013). Preoperative nutrition status and postoperative outcome in elderly general surgery patients: a systematic review. JPEN J Parenter Enteral Nutr.

[141] Lee JL, Oh ES, Lee RW, Finucane TE (2015). Serum albumin and prealbumin in calorically restricted, nondiseased individuals: a systematic review. Am J Med.

[142] Jian G, Lin W, Wang N, Wu J, Wu X (2021). Urine albumin/creatinine ratio and microvascular disease in elderly hypertensive patients without comorbidities. Biomed Res Int.

[143] McGreevy KM, Radak Z, Torma F, Jokai M, Lu AT, Belsky DW, Binder A, Marioni RE, Ferrucci L, Pośpiech E, Branicki W, Ossowski A, Sitek A, Spólnicka M, Raffield LM, Reiner AP, Cox S, Kobor M, Corcoran DL, Horvath S. DNAmFitAge: biological age indicator incorporating physical fitness. Aging (Albany NY). 2023;15(10):3904–38. 10.18632/aging.204538. 10.18632/aging.204538 PMC10258016 36812475

[144] Bernabeu E, McCartney DL, Gadd DA, Hillary RF, Lu AT, Murphy L, Wrobel N, Campbell A, Harris SE, Liewald D, Hayward C, Sudlow C, Cox SR, Evans KL, Horvath S, McIntosh AM, Robinson MR, Vallejos CA, Marioni RE (2023). Refining epigenetic prediction of chronological and biological age. Genome Med.

[145] Chen BH, Marioni RE, Colicino E, Peters MJ, Ward-Caviness CK, Tsai PC, Roetker NS, Just AC, Demerath EW, Guan W, Bressler J, Fornage M, Studenski S, Vandiver AR, Moore AZ, Tanaka T, Kiel DP, Liang L, Vokonas P, Schwartz J, Lunetta KL, Murabito JM, Bandinelli S, Hernandez DG, Melzer D, Nalls M, Pilling LC, Price TR, Singleton AB, Gieger C, Holle R, Kretschmer A, Kronenberg F, Kunze S, Linseisen J, Meisinger C, Rathmann W, Waldenberger M, Visscher PM, Shah S, Wray NR, McRae AF, Franco OH, Hofman A, Uitterlinden AG, Absher D, Assimes T, Levine ME, Lu AT, Tsao PS, Hou L, Manson JE, Carty CL, LaCroix AZ, Reiner AP, Spector TD, Feinberg AP, Levy D, Baccarelli A, van Meurs J, Bell JT, Peters A, Deary IJ, Pankow JS, Ferrucci L, Horvath S (2016). DNA methylation-based measures of biological age: meta-analysis predicting time to death. Aging (Albany NY).

[146] Horvath S, Raj K (2018). DNA methylation-based biomarkers and the epigenetic clock theory of ageing. Nat Rev Genet.

[147] Marioni RE, Shah S, McRae AF, Ritchie SJ, Muniz-Terrera G, Harris SE, Gibson J, Redmond P, Cox SR, Pattie A, Corley J, Taylor A, Murphy L, Starr JM, Horvath S, Visscher PM, Wray NR, Deary IJ (2015). The epigenetic clock is correlated with physical and cognitive fitness in the Lothian Birth Cohort 1936. Int J Epidemiol.

[148] Quach A, Levine ME, Tanaka T, Lu AT, Chen BH, Ferrucci L, Ritz B, Bandinelli S, Neuhouser ML, Beasley JM, Snetselaar L, Wallace RB, Tsao PS, Absher D, Assimes TL, Stewart JD, Li Y, Hou L, Baccarelli AA, Whitsel EA, Horvath S (2017). Epigenetic clock analysis of diet, exercise, education, and lifestyle factors. Aging (Albany NY).

[149] Post Hospers G, Smulders YM, Maier AB, Deeg DJ, Muller M (2015). Relation between blood pressure and mortality risk in an older population: role of chronological and biological age. J Intern Med.

[150] Waaijer ME, Gunn DA, Catt SD, van Ginkel M, de Craen AJ, Hudson NM, van Heemst D, Slagboom PE, Westendorp RG, Maier AB (2012). Morphometric skin characteristics dependent on chronological and biological age: the Leiden longevity study. Age (Dordr).

[151] Crimmins EM, Thyagarajan B, Kim JK, Weir D, Faul J (2021). Quest for a summary measure of biological age: the health and retirement study. Geroscience.

[152] Drewelies J, Hueluer G, Duezel S, Vetter VM, Pawelec G, Steinhagen-Thiessen E, Wagner GG, Lindenberger U, Lill CM, Bertram L, Gerstorf D, Demuth I. Using blood test parameters to define biological age among older adults: association with morbidity and mortality independent of chronological age validated in two separate birth cohorts. Geroscience. 2022;44(6):2685–99. 10.1007/s11357-022-00662-9. 10.1007/s11357-022-00662-9 PMC9768057 36151431

[153] Klemera P, Doubal S (2006). A new approach to the concept and computation of biological age. Mech Ageing Dev.

[154] Kwon D, Belsky DW (2021). A toolkit for quantification of biological age from blood chemistry and organ function test data: BioAge. Geroscience.

[155] Verschoor CP, Belsky DW, Ma J, Cohen AA, Griffith LE, Raina P (2021). Comparing biological age estimates using domain-specific measures from the Canadian longitudinal study on aging. J Gerontol A Biol Sci Med Sci.

[156] Othmani A, Taleb AR, Abdelkawy H, Hadid A (2020). Age estimation from faces using deep learning: a comparative analysis. Comput Vis Image Underst.

[157] Ashiqur Rahman S, Giacobbi P, Pyles L, Mullett C, Doretto G, Adjeroh DA (2021). Deep learning for biological age estimation. Brief Bioinform.

[158] Kivimaki M, Tabak AG, Batty GD, Ferrie JE, Nabi H, Marmot MG, Witte DR, Singh-Manoux A, Shipley MJ (2010). Incremental predictive value of adding past blood pressure measurements to the Framingham hypertension risk equation: the Whitehall II study. Hypertension.

[159] Kivimaki M, Batty GD, Singh-Manoux A, Ferrie JE, Tabak AG, Jokela M, Marmot MG, Smith GD, Shipley MJ (2009). Validating the Framingham hypertension risk score: results from the Whitehall II study. Hypertension.

[160] Tabak AG, Carstensen M, Witte DR, Brunner EJ, Shipley MJ, Jokela M, Roden M, Kivimaki M, Herder C (2012). Adiponectin trajectories before type 2 diabetes diagnosis: Whitehall II study. Diabetes Care.

[161] Jensen AC, Barker A, Kumari M, Brunner EJ, Kivimaki M, Hingorani AD, Wareham NJ, Tabak AG, Witte DR, Langenberg C (2011). Associations of common genetic variants with age-related changes in fasting and postload glucose: evidence from 18 years of follow-up of the Whitehall II cohort. Diabetes.

[162] Carstensen M, Herder C, Kivimaki M, Jokela M, Roden M, Shipley MJ, Witte DR, Brunner EJ, Tabak AG (2010). Accelerated increase in serum interleukin-1 receptor antagonist starts 6 years before diagnosis of type 2 diabetes: Whitehall II prospective cohort study. Diabetes.

[163] Kivimaki M, Tabak AG, Lawlor DA, Batty GD, Singh-Manoux A, Jokela M, Virtanen M, Salo P, Oksanen T, Pentti J, Witte DR, Vahtera J (2010). Antidepressant use before and after the diagnosis of type 2 diabetes: a longitudinal modeling study. Diabetes Care.

[164] Tabak AG, Kivimaki M, Brunner EJ, Lowe GD, Jokela M, Akbaraly TN, Singh-Manoux A, Ferrie JE, Witte DR (2010). Changes in C-reactive protein levels before type 2 diabetes and cardiovascular death: the Whitehall II study. Eur J Endocrinol.

[165] Wacholder S (1991). Practical considerations in choosing between the case-cohort and nested case-control designs. Epidemiology.

[166] Petervari M, Benczik B, Balogh OM, Petrovich B, Agg B, Ferdinandy P (2022). Network analysis for signal detection in spontaneous adverse event reporting database: application of network weighting Normalization to characterize cardiovascular drug safety. Drug Saf.

[167] Ferdinandy P, Baczko I, Bencsik P, Giricz Z, Gorbe A, Pacher P, Varga ZV, Varro A, Schulz R (2019). Definition of hidden drug cardiotoxicity: paradigm change in cardiac safety testing and its clinical implications. Eur Heart J.

[168] Wilkinson MD, Dumontier M, Aalbersberg IJ, Appleton G, Axton M, Baak A, Blomberg N, Boiten JW, da Silva Santos LB, Bourne PE, Bouwman J, Brookes AJ, Clark T, Crosas M, Dillo I, Dumon O, Edmunds S, Evelo CT, Finkers R, Gonzalez-Beltran A, Gray AJ, Groth P, Goble C, Grethe JS, Heringa J, t Hoen PA, Hooft R, Kuhn T, Kok R, Kok J, Lusher SJ, Martone ME, Mons A, Packer AL, Persson B, Rocca-Serra P, Roos M, van Schaik R, Sansone SA, Schultes E, Sengstag T, Slater T, Strawn G, Swertz MA, Thompson M, van der Lei J, van Mulligen E, Velterop J, Waagmeester A, Wittenburg P, Wolstencroft K, Zhao J, Mons B. The FAIR Guiding Principles for scientific data management and stewardship. Sci Data. 2016;3:160018.

[169] Dorey FJ (2011). Statistics in brief: statistical power: what is it and when should it be used?. Clin Orthop Relat Res.

[170] Rothman K, Greenland S, Lash TL. Modern epidemiology. 3rd ed. Lippincott Williams & Wilkins; 2008.

[171] Kim G, Kim H, Yun B, Sim J, Kim C, Oh Y, Yoon J, Lee J. Association of occupational noise exposure and incidence of metabolic syndrome in a retrospective cohort study. Int J Environ Res Public Health. 2022;19(4):2209. 10.3390/ijerph19042209. 10.3390/ijerph19042209 PMC8872108 35206396

[172] Pereira M, Lunet N, Paulo C, Severo M, Azevedo A, Barros H (2012). Incidence of hypertension in a prospective cohort study of adults from Porto. Portugal BMC Cardiovasc Disord.

[173] Wisgerhof W, Ruijgrok C, den Braver NR, Borgonjen-van den Berg KJ, van der Heijden A, Elders PJM, Beulens JWJ, Alssema M. Phenotypic and lifestyle determinants of HbA1c in the general population-The Hoorn Study. PLoS One. 2020;15:e0233769. 10.1371/journal.pone.0233769 PMC7272077 32497119

[174] Pan L, Freedman DS, Gillespie C, Park S, Sherry B (2011). Incidences of obesity and extreme obesity among US adults: findings from the 2009 Behavioral Risk Factor Surveillance System. Popul Health Metr.

[175] Domjan BA, Ferencz V, Tanczer T, Szili-Janicsek Z, Barkai L, Hidvegi T, Jermendy G, Kempler P, Winkler G, Gero L, Tabak AG (2017). Large increase in the prevalence of self-reported diabetes based on a nationally representative survey in Hungary. Prim Care Diabetes.

[176] Jermendy G, Kiss Z, Rokszin G, Abonyi-Toth Z, Wittmann I, Kempler P (2019). Decreasing incidence of pharmacologically treated type 2 diabetes in Hungary from 2001 to 2016: a nationwide cohort study. Diabetes Res Clin Pract.

[177] Sonkodi B, Sonkodi S, Steiner S, Helis E, Turton P, Zachar P, Abraham G, Legrady P, Fodor JG (2012). High prevalence of prehypertension and hypertension in a working population in Hungary. Am J Hypertens.

[178] Veroszta Z, Kopcsó K, Boros J, Kapitány B, Szabó L, Spéder Z (2020). Tracking the development of children from foetal age: an introduction to cohort ’18 Growing Up in Hungary. Longitudinal Life Course Stud.

[179] Borsch-Supan A, Brandt M, Hunkler C, Kneip T, Korbmacher J, Malter F, Schaan B, Stuck S, Zuber S; Team SCC. Data resource profile: the survey of health, ageing and retirement in Europe (SHARE). Int J Epidemiol. 2013;42:992-1001 10.1093/ije/dyt088 PMC3780997 23778574

[180] Batty GD, Shipley M, Tabak A, Singh-Manoux A, Brunner E, Britton A, Kivimaki M (2014). Generalizability of occupational cohort study findings. Epidemiology.

[181] World Health Organization. WHO package of essential noncommunicable (PEN) disease interventions for primary health care. 2020. https://www.who.int/publications/i/item/9789240009226. Geneva.

[182] Damen JA, Hooft L, Schuit E, Debray TP, Collins GS, Tzoulaki I, Lassale CM, Siontis GC, Chiocchia V, Roberts C, Schlussel MM, Gerry S, Black JA, Heus P, van der Schouw YT, Peelen LM, Moons KG (2016). Prediction models for cardiovascular disease risk in the general population: systematic review. BMJ.

[183] Ko DT, Sivaswamy A, Sud M, Kotrri G, Azizi P, Koh M, Austin PC, Lee DS, Roifman I, Thanassoulis G, Tu K, Udell JA, Wijeysundera HC, Anderson TJ (2020). Calibration and discrimination of the Framingham risk score and the pooled cohort equations. CMAJ.

[184] Group Sw and Collaboration ESCCr (2021). SCORE2 risk prediction algorithms: new models to estimate 10-year risk of cardiovascular disease in Europe. Eur Heart J.

[185] Hippisley-Cox J, Coupland C, Brindle P (2017). Development and validation of QRISK3 risk prediction algorithms to estimate future risk of cardiovascular disease: prospective cohort study. BMJ.

[186] Hajifathalian K, Ueda P, Lu Y, Woodward M, Ahmadvand A, Aguilar-Salinas CA, Azizi F, Cifkova R, Di Cesare M, Eriksen L, Farzadfar F, Ikeda N, Khalili D, Khang YH, Lanska V, Leon-Munoz L, Magliano D, Msyamboza KP, Oh K, Rodriguez-Artalejo F, Rojas-Martinez R, Shaw JE, Stevens GA, Tolstrup J, Zhou B, Salomon JA, Ezzati M, Danaei G (2015). A novel risk score to predict cardiovascular disease risk in national populations (Globorisk): a pooled analysis of prospective cohorts and health examination surveys. Lancet Diabetes Endocrinol.

[187] Damen J, Hooft L, Moons KGM (2018). Contemporary cardiovascular risk prediction. Lancet.

[188] Asgari S, Khalili D, Hosseinpanah F, Hadaegh F (2021). Prediction models for type 2 diabetes risk in the general population: a systematic review of observational studies. Int J Endocrinol Metab.

[189] Williams SA, Kivimaki M, Langenberg C, Hingorani AD, Casas JP, Bouchard C, Jonasson C, Sarzynski MA, Shipley MJ, Alexander L, Ash J, Bauer T, Chadwick J, Datta G, DeLisle RK, Hagar Y, Hinterberg M, Ostroff R, Weiss S, Ganz P, Wareham NJ (2019). Plasma protein patterns as comprehensive indicators of health. Nat Med.

[190] Buergel T, Steinfeldt J, Ruyoga G, Pietzner M, Bizzarri D, Vojinovic D, Upmeier Zu Belzen J, Loock L, Kittner P, Christmann L, Hollmann N, Strangalies H, Braunger JM, Wild B, Chiesa ST, Spranger J, Klostermann F, van den Akker EB, Trompet S, Mooijaart SP, Sattar N, Jukema JW, Lavrijssen B, Kavousi M, Ghanbari M, Ikram MA, Slagboom E, Kivimaki M, Langenberg C, Deanfield J, Eils R, Landmesser U. Metabolomic profiles predict individual multidisease outcomes. Nat Med. 2022;28:2309–2320. 10.1038/s41591-022-01980-3 PMC9671812 36138150

[191] Williams SA, Ostroff R, Hinterberg MA, Coresh J, Ballantyne CM, Matsushita K, Mueller CE, Walter J, Jonasson C, Holman RR, Shah SH, Sattar N, Taylor R, Lean ME, Kato S, Shimokawa H, Sakata Y, Nochioka K, Parikh CR, Coca SG, Omland T, Chadwick J, Astling D, Hagar Y, Kureshi N, Loupy K, Paterson C, Primus J, Simpson M, Trujillo NP, Ganz P. A proteomic surrogate for cardiovascular outcomes that is sensitive to multiple mechanisms of change in risk. Sci Transl Med. 2022;14:eabj9625. 10.1126/scitranslmed.abj9625 35385337

[192] Sanghera DK, Bejar C, Sharma S, Gupta R, Blackett PR (2019). Obesity genetics and cardiometabolic health: potential for risk prediction. Diabetes Obes Metab.

[193] de Haan HG, Bezemer ID, Doggen CJ, Le Cessie S, Reitsma PH, Arellano AR, Tong CH, Devlin JJ, Bare LA, Rosendaal FR, Vossen CY (2012). Multiple SNP testing improves risk prediction of first venous thrombosis. Blood.

[194] Piko P, Fiatal S, Kosa Z, Sandor J, Adany R (2017). Genetic factors exist behind the high prevalence of reduced high-density lipoprotein cholesterol levels in the Roma population. Atherosclerosis.

[195] Piko P, Werissa NA, Fiatal S, Sandor J, Adany R (2020). Impact of genetic factors on the age of onset for type 2 diabetes mellitus in addition to the conventional risk factors. J Pers Med.

[196] Fiatal S, Adany R (2017). Application of single-nucleotide polymorphism-related risk estimates in identification of increased genetic susceptibility to cardiovascular diseases: a literature review. Front Public Health.

[197] D'Angelo G, Osol G (1994). Modulation of uterine resistance artery lumen diameter by calcium and G protein activation during pregnancy. Am J Physiol.

[198] Steptoe A, Breeze E, Banks J, Nazroo J (2013). Cohort profile: the English longitudinal study of ageing. Int J Epidemiol.

[199] Hoogendijk EO, Deeg DJ, Poppelaars J, van der Horst M, Broese van Groenou MI, Comijs HC, Pasman HR, van Schoor NM, Suanet B, Thomese F, van Tilburg TG, Visser M, Huisman M. The longitudinal aging study Amsterdam: cohort update 2016 and major findings. Eur J Epidemiol. 2016;31:927–45. 10.1007/s10654-016-0192-0 PMC5010587 27544533

[200] Berk L, van Boxtel M, Kohler S, van Os J (2017). Positive affect and cognitive decline: a 12-year follow-up of the Maastricht aging study. Int J Geriatr Psychiatry.

[201] Rensma SP, van Sloten TT, Houben A, Kohler S, van Boxtel MPJ, Berendschot T, Jansen JFA, Verhey FRJ, Kroon AA, Koster A, Backes WH, Schaper N, Dinant GJ, Schalkwijk CG, Henry RMA, Wolfs EML, van Heumen MJA, Schram MT, Stehouwer CDA (2020). Microvascular dysfunction is associated with worse cognitive performance: the Maastricht study. Hypertension.

[202] Netterstrom B, Kristensen TS, Jensen G, Schnor P (2010). Is the demand-control model still a usefull tool to assess work-related psychosocial risk for ischemic heart disease? Results from 14 year follow up in the Copenhagen City Heart study. Int J Occup Med Environ Health.

[203] UCL Health Survey/Version B, Whitehall II phase 13, Questionnaire. Available here: https://www.ucl.ac.uk/epidemiology-health-care/sites/epidemiology_health_care/files/health_survey_questionnaire_190124_final_1.pdf. Accessed 24 May 2023.

[204] EUROSTAT. European Health Interview Survey (EHIS) (modified Hungarian version). 2018. Available from: https://ec.europa.eu/eurostat/cache/metadata/en/hlth_det_esms.htm. Accessed on 24 May 2023

[205] Balestroni G, Bertolotti G (2012). EuroQol-5D (EQ-5D): an instrument for measuring quality of life. Monaldi Arch Chest Dis.

[206] Ware JE, Kosinski M, Keller SD. SF-36 physical and mental health summary scales: A user's manual. Boston, MA: Health Assessment Lab, New England Medical Center; 1994.

[207] Heatherton TF, Kozlowski LT, Frecker RC, Rickert W, Robinson J (1989). Measuring the heaviness of smoking: using self-reported time to the first cigarette of the day and number of cigarettes smoked per day. Br J Addict.

[208] Saunders JB, Aasland OG, Babor TF, de la Fuente JR, Grant M (1993). Development of the alcohol use disorders identification test (AUDIT): WHO collaborative project on early detection of persons with harmful alcohol consumption–II. Addiction.

[209] Acs P, Veress R, Rocha P, Doczi T, Raposa BL, Baumann P, Ostojic S, Permusz V, Makai A (2021). Criterion validity and reliability of the International Physical Activity Questionnaire - Hungarian short form against the RM42 accelerometer. BMC Public Health.

[210] World Health Organization. Oral health questionnaire for adults. In: Oral health surveys: basic methods, 5th ed. Annex 7.

[211] Maslach C, Jackson SE, Leiter MP. Maslach burnout inventory. In: Evaluating stress: a book of resources. 3rd ed. Lanham: Scarecrow Education; 1997. p. 191–218.

