# Supplementary material for: The Semmelweis Study: a longitudinal occupational cohort study within the framework of the Semmelweis Caring University Model Program for supporting healthy aging
Source: GeroScience. 2023 Dec 7;46(1):191–218. doi: 10.1007/s11357-023-01018-7 (PMC10828351; doi:10.1007/s11357-023-01018-7)
Supplement: Supplementary file 1 — Supplementary file1 (PDF 385 KB) [file 11357_2023_1018_MOESM1_ESM.pdf]

SEMMELWEIS STUDY

PROSPECTIVE OCCUPATIONAL COHORT STUDY OF THE SEMMELWEIS UNIVERSITY

SAMPLE

UNIVERSITAS BUDAPESTINENSIS DE SEMMELWEIS NOMINATA 1769

TKP2021-NKTA-47

QUESTIONNAIRE

Table of Contents

Sociodemographic data 3
Subjective health 15
Nutrition 16
Oral health 23
Smoking, e-cigarettes, and heated tobacco products 24
Alcohol consumption 26
Illegal and prescription drug abuse 27
Stress 28
Relationship 29
Physical activity 30
Sleep 31
Cognitive abilities 32
References 33

SAMPLE

Sociodemographic data

1. Do you work at Semmelweis University?
    a. Yes, at the moment
    b. Yes, in the past
    c. I don't and I never did

2. What is your sex?
    a. Male
    b. Female
    c. Trans male
    d. Trans female
    e. Other, please specify:................................

3. Are you pregnant at the moment?
    a. Yes
    b. No

4. Which year were you born in?
    a. …………………

5. What is your weight (kg)?
    a. …………………

6. What is your height (cm)?
    a. …………………

7. What shoe size do you wear?

    a. …………………

8. What is your eye color?
    a. Albino
    b. Light blue

SAMPLE

c. Dark blue
d. Light brown
e. Dark brown
f. Green
g. Brownish green
h. Brownish blue
i. Greenish blue
j. Other, please specify………….

9. What is your ethnicity/nationality?
   a. Hungarian
   b. Romani
   c. Bulgarian
   d. Greek
   e. Polish
   f. German
   g. Armenian
   h. Ruthenian
   i. Serbian
   j. Slovenian
   k. Ukranian
   l. Arabic
   m. Chinese
   n. Vietnamese
   o. Russian
   p. Other, please specify:………..

10. Do you consider yourself religious?
    a. Yes
    b. Yes, in my own may
    c. No

11. If you are religious, do you actively take part in the life of your religious community?
    a. Yes, regularly
    b. Yes, occasionally

SAMPLE

c. No

12. Where do you live?
    a. …………….

13. What is the highest level of your attained education?
    a. Less than primary
    b. Primary
    c. Secondary
    d. Tertiary

14. How many total years of education did you take part in?
    a. ....

15. What is your job title?
    a. ....

16. What percentage of your time do you spend sitting down?
    a. ....

17. What type of labor contract did you sign?
    a. Indefinite
    b. Definite
    c. Occasional agreement
    d. Other, please specify:……….

18. Which one of these describes your main job?
    a. I work 36 hours a week
    b. I have a main and a side job
    c. I work part time (<36 hours)
    d. I work, but I am retired
    e. Medical intern
    f. Student
    g. Other, please specify:……….

SAMPLE

19. Officially how many hours do you work a week?

    a. ....

20. How many days do you work in a month?

    a. ....

21. How many hours a day do you spend at work on average?

    a. ....

22. In a typical month, how many days do you work on weekends?

    a. ....

23. In a typical month, how many night shifts do you have?

    a. ....

24. Is your job flexible?

    a. Yes
    b. Partially
    c. No

25. On a scale of 1–10 where 1 is the lowest social class and 10 is the highest, where do you position yourself within the Hungarian society?

    a. ....

26. How much do you earn in a month?

    a. <200 000Ft
    b. 200 000–349 999Ft
    c. 350 000–599 999Ft
    d. 600 000–799 999Ft
    e. 800 000–999 999
    f. 1 million Ft or more
    g. I don’t know
    h. I don’t want to answer

SAMPLE

27. Based on your income, on a scale of 1–10 where 1 is the lowest income and 10 is the highest, where do you position yourself within the Hungarian society?

    a. ....

The following four questions are the questions of the Copenhagen City Heart Study (1)

28. Are you worried that you will become unemployed?

    a. Yes
    b. No

29. Are you worried that you will be reassigned to a different job?

    a. Yes
    b. No

30. Are you worried that your job will become superfluous because of new technologies?

    a. Yes
    b. No

31. Are you worried that it will be hard for you to find a new job with your current qualifications?

    a. Yes
    b. No

32. If you are in retirement, why did you decide to continue working?

    a. Enjoyment of job
    b. Financially to add to my income
    c. Financially I cannot afford to retire
    d. For social contact
    e. To keep active
    f. You feel you ought to continue working
    g. Your spouse/partner is working

33. How many people live in your household?

    a. ....

SAMPLE

34. How many individuals less than 18 years of age live in your household?

    a. ………

35. How many adults contribute to your total family income?

    a. ………

36. How much is your or your family's total income?

    a. <200 000Ft
    b. 200 000–349 999Ft
    c. 350 000–599 999Ft
    d. 600 000–799 999Ft
    e. 800 000–999 999Ft
    f. 1–1 999 million Ft
    g. 2 million Ft or above
    h. I don't know
    i. I don't want to answer

37. How many months' worth of savings do you have?

    a. Less than 1 month's
    b. 1–2 months'
    c. 3–6 months'
    d. 7–12 months'
    e. More than a year's

38. How much savings do you have?

    a. A significant amount
    b. Some savings
    c. No savings
    d. I don't want to answer

39. How much mortgage do you have?

    a. I have a significant amount
    b. I have some
    c. I don't have any

SAMPLE

d. I don’t want to answer

40. Where do you live at the moment?
    a. In my own apartment/house
    b. I rent an apartment/house
    c. I live in someone else’s apartment/house without paying rent
    d. Other

41. Do you or your spouse/partner own any additional properties apart from the apartment/house you presently live in?
    a. Yes, 1 additional property
    b. Yes, 2 additional properties
    c. Yes, 3 or more additional properties
    d. None

42. How many cars does your household own?
    a. None
    b. 1
    c. 2
    d. 3 or more

Are you affected or have you been told by a doctor that you are affected from the following conditions?

	No	Yes	If yes, when?
43. Stroke	0	1	
44. Transient ischemic attack	0	1	
45. Other cerebrovascular disease (please specify)	0	1	
46. Angina	0	1	
47. Acute myocardial infarction	0	1	
48. Heart failure	0	1	
49. Hypertension	0	1	
50. Arrhythmia	0	1	
51. Deep vein thrombosis	0	1	
52. Other cardiovascular disease (please specify)			
53. Diabetes	0	1	
a. Diabetes Type 1	0	1	

SAMPLE

b. Diabetes Type 2	0	1	
54. Cancer (please specify)	0	1	
55. Chronic obstructive lung disease	0	1	
56. Other lung disease (please specify)			
57. Irritable bowel syndrome/disease	0	1	
58. Other gastrointestinal disease (please specify)	0	1	
59. Central nervous system disease (please specify)	0	1	
60. Musculoskeletal disease (please specify)	0	1	
61. Mental disease (please specify)	0	1	
62. Autoimmune disease (please specify)	0	1	
63. Thyroid disease	0	1	
64. Other disease (please specify)	0	1	
65. COVID-19	0	1	
66. Post-COVID, Long-COVID	0	1	

67. Have you ever taken part in an exercise electrocardiogram?
    a. Yes
    b. No
    c. If yes, when?

68. Have you ever taken part in an angiogram or an X-ray of your coronaries?
    a. Yes
    b. No
    c. If yes, when?

69. Have they ever performed angioplasty of your coronaries on you?
    a. Yes
    b. No
    c. If yes, when?

70. Have you ever had a coronary artery bypass graft?
    a. Yes
    b. No
    c. If yes, when?

SAMPLE

71. Have you been ever admitted to a hospital because of chest pain/angina/acute myocardial infarction?

   a. Yes
   b. No
   c. If yes, when?

72. Have you ever taken part in any other examination of your heart or vessels?

   a. Yes
   b. No
   c. If yes, when?

73. Do your legs hurt while walking?

   a. Yes
   b. No
   c. If yes, how many meters does it take for the pain to appear?

74. How limited are you in your physical activity?

   a. No limitation of physical activity. Ordinary physical activity does not cause undue fatigue, palpitation, dyspnea (shortness of breath).
   b. Slight limitation of physical activity. Comfortable at rest. Ordinary physical activity results in fatigue, palpitation, dyspnea (shortness of breath).
   c. Marked limitation of physical activity. Comfortable at rest. Less than ordinary activity causes fatigue, palpitation, or dyspnea.
   d. Unable to carry on any physical activity without discomfort. If any physical activity is undertaken, discomfort increases.

If you are a male, then skip the following questions

75. How old were you at your first menstruation?

   a. ....

76. How old were you at your last menstruation?

   a. ....
   b. I am still menstruating

SAMPLE

77. Were you ever pregnant?

   a. Yes
   b. No

78. How many times were you pregnant?

   a. ....

79. How many pregnancies ended in live births?

   a. ....

80. Are you affected or have you been ever told that you are affected by endometriosis?

   a. Yes
   b. No

81. Are you affected or have you been ever told that you are affected by polycystic ovary syndrome?

   a. Yes
   b. No

82. Have you ever taken part in any assistive reproduction procedures?

   a. No
   b. Yes, in insemination, which resulted in ..... number of live births
   c. Yes, in *in in vitro* fertilization, which resulted in ..... number of live births

83. Have you ever had an induced abortion?

   a. Yes
   b. No

84. Have you ever had a spontaneous abortion?

   a. Yes
   b. No

85. Are you affected or have you been ever told that you are affected by gestational hypertension?

   a. Yes

SAMPLE

b. No

86. Are you affected or have you been ever told that you are affected by gestational diabetes?

a. Yes

b. No

87. Are you affected or have you been ever told that you are affected by preeclampsia?

a. Yes

b. No

88. What medication(s) are you on?

Name	Dose	Name	Dose

89. Is your mother still alive?

a. Yes

b. No

c. Not applicable

90. If your mother is alive, how old is she?

a. ....

91. If your mother is not alive, how old was she when she passed away?

a. ....

92. Is/was your mother active above 75 years of age?

a. ....

93. Is your father still alive?

a. Yes

b. No

SAMPLE

c. Not applicable

94. If your father is alive, how old is he?

a. ....

95. If your father is not alive, how old was he when he passed away?

a. ....

96. Is/was your father active above 75 years of age?

a. ....

97. How many siblings do you have?

a. ....

What kind of diseases occurred in your family?

	Mother		Father		Sibling	
	No	Yes	No	Yes	No	Yes
98. Acute myocardial infarction	0	1	0	1	0	1
99. Stroke	0	1	0	1	0	1
100. Deep vein thrombosis	0	1	0	1	0	1
101. Pulmonary emboly	0	1	0	1	0	1
102. Hypertension	0	1	0	1	0	1
103. Any form of cancer	0	1	0	1	0	1
104. Diabetes	0	1	0	1	0	1
105. Thyroid disease	0	1	0	1	0	1
106. Chronic lung disease	0	1	0	1	0	1
107. Autoimmune disease	0	1	0	1	0	1
108. Mental disease	0	1	0	1	0	1
109. Dementia	0	1	0	1	0	1

SAMPLE

Subjective health

SF-36 (2)

EuroQoL-5D-3L (3)

1. Has your health changed compared to your health before the COVID-19 pandemic?
   0) It improved
   1) It did not change
   2) It deteriorated
   3) I don't know

SAMPLE

Nutrition

Now we are going to ask you questions about your eating and drinking habits in the past 12 months.

1. How often do you consume fresh, frozen, dried, or canned fruit?
    a. More than once per day
    b. Once per day
    c. 4-6 times a week
    d. 1-3 times a week
    e. Monthly
    f. Never

2. On days when you consume fresh, frozen, dried, or canned fruit, how many portions do you usually consume? [1 portion = 10 dkg fruit (for example a medium sized apple or orange) or a cup of berries]
    a. .... portion

3. How often do you consume fresh or raw fruit?
    a. More than once per day
    b. Once per day
    c. 4-6 times a week
    d. 1-3 times a week
    e. Monthly
    f. Never

4. On days when you consume fresh or raw fruit, how many portions do you usually consume? [1 serving = 10 dkg fruit (for example a medium sized apple or orange) or a cup of berries]
    a. ..... portions

5. How often do you consume fresh, frozen, dried, or canned vegetables or salad (excluding potatoes)?
    a. More than once per day
    b. Once per day
    c. 4-6 times a week
    d. 1-3 times a week
    e. Monthly
    f. Never

SAMPLE

6. On days when you consume fresh, frozen, dried, or tinned vegetables or salad (excluding potato) how many portions do you usually consume? [1 serving = 10 dkg fresh, steamed, cooked, or roasted seasonal vegetables (for example 1 medium sized pepper, tomato) or 1 little plate of salad.]
   a. ..... portions

7. How often do you consume fresh or raw vegetables?
   a. More than once per day
   b. Once per day
   c. 4-6 times a week
   d. 1-3 times a week
   e. Monthly
   f. Never

8. On days when you consume fresh or raw vegetables, how many portions do you usually consume? [1 serving = 10 dkg fresh, steamed, cooked, or roasted seasonal vegetables (for example 1 medium sized pepper, tomato) or 1 little plate salad]
   a. .... portions

9. How often do you consume legumes/pulses (peas, beans, lentils, chickpeas etc.)?
   a. More than once per day
   b. Once per day
   c. 4-6 times a week
   d. 1-3 times a week
   e. Monthly
   f. Never

10. On days when you consume legumes/pulses (peas, beans, lentils, chickpeas etc.), how many portions do you usually consume?
   a. ..... portions

11. How often do you consume nuts and oilseeds (e.g., walnut, almond, hazelnut, peanut, pumpkin seed, sunflower seed)?
   a. More than once per day

SAMPLE

b. Once per day
c. 4-6 times a week
d. 1-3 times a week
e. Monthly
f. Never

12. On days when you consume oilseeds or nuts, how many servings do you take? (1 serving = 30g, approximately a cup of your hand of walnut, almond, hazelnut, peanut, pumpkin seed, sunflower seed)
    a. ..... portions

13. How much water do you drink on an average day?
    a. at least 2 liters
    b. more than 1.5 liters but less than 2 liters
    c. 1 liter or more than 1 liter, but less than 1.5 liters
    d. 0.5 liter or more, but less than 1 liter
    e. less than 0.5 liter
    f. I do not drink water daily or at all

14.	How often do you drink the following?	Daily	4-6 times a week	1-3 times a week	Monthly	Never	Daily
14/A	100% fruit juice						
14/B	Sugary soft drinks						
14/C	Sugar free soft drinks						
14/D	Energy drinks						

15. On days when you consume 100% fruit juice, how much do you consume?
    a. 500 ml or more
    b. 330-500 ml
    c. 251-330 ml
    d. 151-250 ml
    e. 150 ml or less
    f. I don't consume these types of drinks

16. On days when you consume sugary soft drinks, how much do you consume?

SAMPLE

a. 500 ml or more
b. 330-500 ml
c. 251-330 ml
d. 151-250 ml
e. 150 ml or less
f. I don't consume these types of drinks

17. On days when you consume sugar free soft drinks, how much do you consume?
    a. 500 ml or more
    b. 330-500 ml
    c. 251-330 ml
    d. 151-250 ml
    e. 150 ml or less
    f. I don't consume these types of drinks

18. On days when you consume energy drinks, how much do you consume?
    a. 500 ml or more
    b. 330-500 ml
    c. 251-330 ml
    d. 151-250 ml
    e. 150 ml or less
    f. I don't consume these types of drinks

19.	How often do you drink the following?	Tea	Coffee	Coco
	a. Daily, more than once			
	b. Once a day			
	c. 4-6 times a week			
	d. 1-3 times a week			
	e. Monthly			
	f. Never			
20.	When you drink tea, coffee or coco, do you add any sweeteners to your drink?	Tea	Coffee	Coco
	a. Yes, mainly natural sweeteners			
	b. Yes, mainly artificial sweeteners			

SAMPLE

	c. I don’t use any form of sweetener			

21. How often do you consume deserts or sweets?
    a. Daily, more than once
    b. Once a day
    c. 4-6 times a week
    d. 1-3 times a week
    e. Monthly
    f. Never

22. On days when you consume desert or sweets, how much do you consume? [1 portion=half a bar of chocolate (approx. 5dkg), 3 cookies, one scoop of ice-cream]
    a. ..... portions

23.	How often do you consume the following products?	Daily	4-6 times a week	1-3 times a week	Monthly	Never
23/A	Red meat (e.g., beef, pork)					
23/B	White meat (e.g., poultry)					
23/C	Cold cuts or Processed meat					
23/D	Seafood					

24. How often do you consume dairy products?
    a. Daily, more than once
    b. Once a day
    c. 4-6 times a week
    d. 1-3 times a week
    e. Monthly
    f. Never

25. On days when you consume dairy products, how much do you consume? (1 portion = 2 dl of milk/yoghurt, 3 dkg cheese)
    a. ...... portions

SAMPLE

26. How often do you consume wheat?
    a. Daily, more than once
    b. Once a day
    c. 4-6 times a week
    d. 1-3 times a week
    e. Monthly
    f. Never

27. On days when you consume wheat, how much wheat do you consume? (1 portion = medium sized slice of bread, 20 dkg of pasta, 3 tbl spoons of cereal)
    a. ...... portions

28. How many of these portions contain whole grain wheat? (1 portion = medium sized slice of bread, 20 dkg of pasta, 3 tbl spoons of cereal)
    a. ...... portions

28.	How often do you consume meat prepared with the following methods?	Daily	4-6 times a week	1-3 times a week	Monthly	Never
28/A	Parboiled					
28/B	Deep fried					
28/C	Fried					
28/D	Grilled					

29. When preparing foods, how often do you use olive oil?
    a. I never use olive oil
    b. Seldom
    c. Sometimes
    d. I use olive oil to prepare most foods
    e. I always use olive oil

30. How much margarine or butter do you consume a day (1 portion=12 g butter or margarine)?
    a. Daily, one portion or more
    b. Daily, but less than one portion

SAMPLE

c. 4-6 portions per week
d. 2-3 portions per week
e. Weekly one portion or less
f. I don't consume butter or margarin

31. Do you add salt to your food once it's done, even if salt was added to it during the preparation?
a. Never
b. Sometimes
c. Often
d. Always

32. Do you follow any special form of diet?
a. Diabetic diet
b. Gluten-free die
c. Lactose-free diet
d. Milk-free diet
e. Low energy diet
f. Low sodium diet
g. Vegetarian
h. Other: ……………………………………………………….
i. I don't follow any diet

33. What kind of supplements have you taken in the past week?
a. Vitamin D
b. Vitamin C
c. Omega-3
d. Multivitamin
e. Protein powder
f. Fiber
g. Collagen
h. Other: ………………………

SAMPLE

Oral health

1. How many natural teeth do you have?

a. None

b. 1-9

c. 10-19

d. 20 or more

2. Do you have any tooth replacement dentures?

a. Removable partial denture

b. Upper full removable denture

c. Lower full removable denture

3. When was the last time you visited a dentist?

a. In the past 6 months

b. 6–12 months ago

c. 1–2 years ago

d. 2–5 years ago

e. More than 5 years ago

f. I've never been to the doctor

4. How would you describe the health of your gum and teeth?

	Gum	Teeth
Perfect		
Very good		
Good		
Average		
Bad		
Very bad		
I don't know		

SAMPLE

Smoking, e-cigarettes, and heated tobacco products

The following questions concern smoking, the usage of e-cigarettes and similar electronic devices (e.g. e-shisha), and heated tobacco products (e.g. IQOS). Please note, that under the term ’smoking’, we mean the consumption of manufactured cigarettes or hand-rolled cigarettes.

1. How often do you smoke?
   a. Daily
   b. Weekly
   c. Monthly
   d. Less often than monthly
   e. I have quit smoking.
   f. I have never smoked.
   g. I do not know.
   h. I do not want to answer.

2. On days when you use cigarettes, how many cigarettes do you usually smoke in a day?
   a. __________ cigarettes per day

3. On days when you smoke, how long after waking up do you usually light your first cigarette?
   a. ≥121 minutes
   b. 61-120 minutes
   c. 31-60 minutes
   d. 16-30 minutes
   e. 6-15 minutes
   f. 0-5 minutes

4. How many years have you been smoking daily or almost daily?
   a. …………… year/years
   b. I do not smoke/have smoked daily or almost daily.
   c. I do not know.
   d. I do not want to answer.

5. How long have you not been smoking?

SAMPLE

a. I have not smoked for ___ years and _____ months.

6. Think about the time when you smoked. On days when you smoked, how many cigarettes did you usually smoke a day?

   a. _____ cigarettes per day

7. How often do you currently use e-cigarettes?

   a. Daily.
   b. Weekly
   c. Monthly
   d. Less often than monthly
   e. I have already stopped using it.
   f. I have never used it.
   g. I do not know.
   h. I do not want to answer.

8. How often do you currently use any heated tobacco products, e.g., IQOS?

   a. Daily
   b. Weekly
   c. Monthly
   d. Less often than monthly
   e. I have already stopped using it.
   f. I have never used it.
   g. I do not know.
   h. I do not want to answer.

SAMPLE

Alcohol consumption

10-item Alcohol Use Disorders Identification Test (4)

SAMPLE

Illegal and prescription drug abuse

1. Have you ever used any drugs, or have you ever taken certain prescription drugs in excessive amounts and/or not prescribed by a doctor?

	No	Yes, at least once in my life	Yes, in the last year	Yes, in the last one month
a. Illegal drug use	0	1	2	3
b. Prescription drug abuse	0	1	2	3

SAMPLE

Stress

Perceived Stress Scale (5)

Connor Davidson Brief Resilience Scale (6)

Effort-Reward Imbalance (7)

Maslach Burnout Inventory (8)

SAMPLE

Relationship

1. What is your sexual orientation?
   a. heterosexual
   b. homosexual
   c. bisexual
   d. other: ....

2. What is your marital status (legally)?
   a. unmarried
   b. married
   c. registered partnership/civil union
   d. divorced
   e. widowed

3. Are you currently in a permanent relationship?
   a. No, I do not have a partner
   b. I have short relationships; they cannot be considered permanent
   c. Yes, but we do not live together
   d. Yes, and we live together
   e. Yes, I have a spouse

Stockholm Marital Stress Scale (9)

Relationship Structure Questionnaire (10)

SAMPLE

Physical activity

International Physical Activity Questionnaire Long-form (11)

SAMPLE

Sleep

Pittsburgh Sleep Quality Index (12)

SAMPLE

Cognitive abilities

5-item abbreviation of the International Cognitive Ability Resource (13)

SAMPLE

References

1. Netterstrøm B, Kristensen TS, Jensen G, Schnor P. Is the demand-control model still a usefull tool to assess work-related psychosocial risk for ischemic heart disease? Results from 14 year follow up in the Copenhagen City Heart study. Int J Occup Med Environ Health 2010;23:217-224
2. Ware JE, Kosinski MA, Keller SD. *SF-36 physical and mental health summary scales a user's manual*. Boston, Mass, Health Assessment Lab, New England Med. Center, 1997
3. Balestroni G, Bertolotti G. [EuroQol-5D (EQ-5D): an instrument for measuring quality of life]. Monaldi Arch Chest Dis 2012;78:155-159
4. Saunders JB, Aasland OG, Babor TF, de la Fuente JR, Grant M. Development of the Alcohol Use Disorders Identification Test (AUDIT): WHO Collaborative Project on Early Detection of Persons with Harmful Alcohol Consumption--II. Addiction 1993;88:791-804
5. Cohen S, Kamarck T, Mermelstein R. A global measure of perceived stress. J Health Soc Behav 1983;24:385-396
6. Connor KM, Davidson JR. Development of a new resilience scale: the Connor-Davidson Resilience Scale (CD-RISC). Depress Anxiety 2003;18:76-82
7. Stanhope J. Effort–Reward Imbalance Questionnaire. Occupational Medicine 2017;67:314-315
8. Maslach C, Jackson SE, Leiter MP. Maslach Burnout Inventory (3rd ed.). 1996;
9. Orth-Gomér K, Wamala SP, Horsten M, Schenck-Gustafsson K, Schneiderman N, Mittleman MA. Marital Stress Worsens Prognosis in Women With Coronary Heart DiseaseThe Stockholm Female Coronary Risk Study. JAMA 2000;284:3008-3014
10. Chris Fraley R, Niedenthal PM, Marks M, Brumbaugh C, Vicary A. Adult Attachment and the Perception of Emotional Expressions: Probing the Hyperactivating Strategies Underlying Anxious Attachment. Journal of Personality 2006;74:1163-1190
11. Craig CL, Marshall AL, Sjöström M, Bauman AE, Booth ML, Ainsworth BE, Pratt M, Ekelund U, Yngve A, Sallis JF, Oja P. International physical activity questionnaire: 12-country reliability and validity. Med Sci Sports Exerc 2003;35:1381-1395
12. Buysse DJ, Reynolds CF, Monk TH, Berman SR, Kupfer DJ. The Pittsburgh sleep quality index: A new instrument for psychiatric practice and research. Psychiatry Research 1989;28:193-213
13. Kirkegaard EOW, Bjerrekær J. ICAR5: design and validation of a 5-item public domain cognitive ability test. Open Differential Psychology 2016;

SAMPLE
